# New Benzimidazole-Based pH-Sensitive Fluorescent Probes

**DOI:** 10.3390/molecules30234622

**Published:** 2025-12-01

**Authors:** Artem D. Pugachev, Ivan N. Bardasov, Shorena K. Karchava, Tatiana N. Azhogina, Maria V. Klimova, Alexey E. Matukhno, Vitaly S. Dmitriev, Gennady S. Borodkin, Olga D. Lanovaya, Diana Y. Pobedinskaya, Angelina E. Polinichenko, Ludmila E. Khmelevtsova, Ivan S. Sazykin, Marina A. Sazykina, Ilya V. Ozhogin

**Affiliations:** 1 Institute of Physical and Organic Chemistry, Southern Federal University, Stachki Prosp., 194/2, Rostov-on-Don 344090, Russialanovaia@sfedu.ru (O.D.L.);; 2 Faculty of Chemistry and Pharmaceutics, Chuvash State University Named After I.N. Ulyanov, Moscow Prosp., 15, Cheboksary 428015, Russia; bardasov.chem@mail.ru; 3 Academy of Biology and Biotechnologies, Southern Federal University, Stachki Prosp., 194/1, Rostov-on-Don 344090, Russialehmelevcova@sfedu.ru (L.E.K.);; 4 Research Center for Neurotechnology, Southern Federal University, 194 Stachka Ave., Rostov-on-Don 344090, Russia; 5 Department of Chemistry, North-Caucasus Federal University, Pushkin Street, 1, Stavropol 355017, Russia

**Keywords:** benzimidazole, fluorescence, pH, molecular docking, sulfonic acids, bioimaging, whole-cell lux-biosensors

## Abstract

This article is devoted to the synthesis and investigation of a family of new benzimidazole compounds with a propylsulfonate moiety, synthesized by condensation of salicylic aldehyde or its 5-substituted derivatives with 3-(2,3-dimethylbenzimidazol-1-ium-1-yl)propane-1-sulfonate. The structure of the obtained dyes was confirmed using NMR, FT-IR, and HRMS. Absorption and photoluminescence properties were studied in phosphate buffers over a wide pH range, and changes in the absorption and fluorescence spectra of DMSO solutions upon titration with DIPEA and HCl were also studied. It was found that all the target compounds possess pH-sensitive optical properties and can be used as fluorescent probes, while methoxycarbonyl-substituted derivative **3c** demonstrated the most prominent optical and fluorescent response starting from pH ~ 4.5. The toxicity of the compounds was studied using whole-cell bioluminescent bacterial sensors. The effect on the biomass and metabolic activity of strains *Staphylococcus aureus* ATCC 6538-P FDA 209-P and *Escherichia* CDC F-50 bacterial biofilms was also investigated. In the final stage of the study, bioimaging experiments were carried out using the selected most promising dye **3c** and biofilms. It was demonstrated that the dye can be excited by light with wavelengths of 458 nm or 750 nm in multiphoton mode. Importantly, when biofilms are incubated in the dye solution for 3 h, only the extracellular matrix is stained. However, if the staining time is increased to 24 h, dye penetration into bacterial cells is observed, resulting in a second photoluminescence maximum during sample analysis. It is important to note that when biofilms are incubated in a dye solution for 3 h, only the extracellular matrix is stained, while with longer staining, penetration of the dye into bacterial cells is observed, and a second photoluminescence maximum appears during sample analysis. The results obtained demonstrate a high potential of using benzimidazole-based compounds as pH-sensitive fluorescent probes operating in a biologically relevant pH range, which can be used for imaging of bacterial biofilms.

## 1. Introduction

Intracellular pH plays a vital role in regulating key cellular functions and processes, such as proliferation and apoptosis [1,2,3], ion transport [4,5,6], development of multidrug resistance [7,8], and endocytosis [9,10]. Deviations from normal intracellular pH values are associated with various pathological conditions, including cancer [11] and Alzheimer’s disease [12]. Within cells, reduced pH can trigger critical cellular events, such as protein denaturation or activation of enzymes that are normally inactive at neutral pH values. Therefore, pH measurement provides critical information on the functional state of the cell and pathological changes. Another striking example is the acidic environment of lysosomes (pH 4.5–5.5) [13], which is essential for efficient protein degradation. While it is possible to determine lysosomal pH using biosensors [14], fluorescent pH-sensitive probes offer a powerful alternative.

The demand for accurate pH determination is driving extensive research, particularly for monitoring biological systems and the environment. Fluorescence analysis is recognized as one of the most promising tools in this field [15,16,17]. Compared to alternative methods (microelectrodes, nuclear magnetic resonance (NMR), absorption spectroscopy), fluorescence spectroscopy offers several significant advantages: excellent spatial and temporal resolution, high sensitivity with appropriate probe selection for each specific case, operational simplicity, and, crucially, non-invasiveness.

Two primary types of fluorescent probes exist. The first one exhibits a sharp “on-off” or “off-on” change in fluorescence intensity depending on pH [18]. The second type consists of ratiometric probes, which are characterized by a differential response to pH at two excitation or emission wavelengths [19]. Despite their utility, many existing probes suffer from significant limitations, including photobleaching, minor spectral shifts with changes in pH, suboptimal excitation/emission wavelengths for biological applications, cellular leakage, and low absorption or quantum yield under certain conditions [20,21]. Therefore, the development of new and more advanced intracellular pH indicators remains a pressing task.

Benzimidazole derivatives serve as a versatile platform for constructing fluorescent probes. A bright example is the BH1L probe, specifically optimized for targeting lysosomes and providing direct pH assessment within lysosomal compartments in real time. This technique demonstrates versatility, being applicable both to studies on living cells and to visualization in living mouse brain tissue using two-photon microscopy [22]. Numerous ratiometric probes for ion detection [23,24] and pH determination [22,25,26] have been developed based on the benzimidazole scaffold. The application of such probes for measuring pH within bacterial biofilms is also of significant interest, as it is crucial for understanding the mechanisms underlying their resistance to antimicrobial drugs [27]. In previous work, we demonstrated the feasibility of this approach by visualizing bacterial biofilms of *E. coli* and *A. calcoaceticus* using indoline-based spiropyran salts [28].

Spiropyrans are currently widely used in bioimaging for detecting various ions [29,30], creating drug delivery systems [31], and enabling light- and pH-controlled capture of glycopeptides [32]. However, classical indoline spiropyrans are less suitable for pH visualization, since their protonated forms often fail to bind effectively to biological objects and demonstrate weak fluorescence, while their unprotonated states formed upon exposure to bases are usually not fluorescent on their own [33]. This limitation can be overcome through structural modification. For instance, introducing carboxyl groups can confer a fluorescent response in alkaline media [34]. Similarly, spiropyrans with an alkyl sulfonate fragment at the nitrogen atom, known as metastable-state photoacids [35,36], are highly promising for detecting and controlling biological objects [37,38] and capable of demonstrating a strong fluorescent response in the presence of bases [39].

Most known phthalein, cyanine, and other dyes function in an inappropriate pH range for studying biological samples, particularly bacterial biofilms. Their pKa values are typically greater than 8.5 or less than 5 [40,41]. Many are even designed for extreme pH values [42]. For example, Schlafer et al. visualized bacterial biofilms using the C-SNARF-4 dye, but for most strains, it was found to function only at pH values less than 5.5 [43]. However, some dyes are known that function in the required pH range and have pKa values of 6.4 [44]. However, one of their drawbacks is the expensive, multi-step synthesis, as well as the lack of understanding of their toxicity and their applicability for biological research.

Thus, the current work was aimed at the synthesis of a series of benzimidazole-based merocyanine analogs of spiropyrans with pH-responsive optical properties by simple condensation reaction from inexpensive reagents. To characterize them, it is necessary to carry out comprehensive structural and spectral analysis, biological studies on bioluminescent bacterial sensors and bacterial biofilms, and the investigation of their potential application for pH-imaging of bacterial biofilms.

## 2. Results and Discussion

### 2.1. Molecular Design, Synthesis, and Structure Studies

In this study, a new series of small-molecule benzimidazolium derivatives was synthesized and characterized, which may prove promising for pH determination in cells and bacterial biofilms. The benzimidazolium heterocycle was chosen as the molecular core due to its potentially high probability of binding to bacterial DNA [45], as well as its antibacterial activity [46] using known compounds (e.g., stellanin [47,48]) as an example. The introduction of a propanesulfonate group into the heterocyclic fragment provided two critical advantages: a significant increase in water solubility and an electron-withdrawing effect on the electron system of the molecule. To tune the spectroscopic characteristics, we introduced a methoxycarbonyl or methoxy substituent into position 5′ or left this position unsubstituted. The methoxy substituent, typically acting as a strong electron donor, significantly enhances intramolecular charge transfer [49]. This can be manifested in an increase in the extinction coefficient, fluorescence quantum yield, and a more pronounced shift in the emission spectrum with a change in pH, although its moderate lipophilicity only facilitates passive penetration into cells without specific organelle targeting [50]. The methoxycarbonyl fragment is a special case, since its electron-withdrawing effect can cause a bathochromic shift and affect pKa. Its moderate hydrophilicity balances solubility and membrane permeability [51]. However, the main value of this group is its bioactivation: intracellular hydrolysis by esterases to carboxylate leads to “metabolic retention” of the charged form of the probe inside the cell [52].

**Scheme 1:**
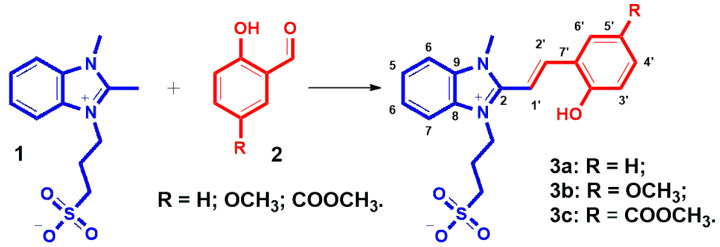
Synthesis of benzimidazolium derivatives **3a**–**c** and numbering of atoms for NMR spectra correlations.

Thus, we synthesized a series of three compounds: 5′-unsubstituted compound **3a**, 5′-methoxy derivative **3b**, and 5-methoxycarbonyl-substituted compound **3c**. All of them were obtained by condensation of 3-(2,3-dimethylbenzimidazol-1-ium-1-yl)propane-1-sulfonate **1** with the corresponding salicylaldehyde derivative **2** (1.1 equiv) in methanol under heating. The reaction was catalyzed by a small amount of organic base (Scheme 1). It should be noted that the synthesis of the compound with an electron-withdrawing substituent gave the highest product yield (77.4%), while the reaction with the methoxy-substituted salicylic aldehyde proceeded with the lowest yield of 43.5%. The yield for the unsubstituted derivative **3a** was intermediate −51.1%. The electron-donating substituent likely slightly reduces the activity of the formyl group of the salicylic aldehyde derivative for this reaction. The effect of the electron-withdrawing substituent on reactivity was the opposite, and the yield of the product in our case increased by more than 25%.

Molecular structure of compounds **3a**–**c** was confirmed using NMR and FTIR spectroscopy, as well as HRMS. All NMR spectra correspond to the proposed structures in terms of chemical shifts, integrated intensities, and spin–spin interaction constants. All the signals were assigned using two-dimensional and heteronuclear NMR spectroscopy techniques.

In the ^1^H NMR spectra of all compounds, the signals of the –OH group were detected, indicating the localization of the negative charge on the sulfonyl group, as in metastable photoacids based on indoline heterocycle [35,36]. These singlet signals are located at 10.46, 9.97 and 11.55 ppm for **3a**, **3b** and **3c**, respectively. In the ^13^C NMR spectrum, the signals of the carbon atoms of the C–OH groups were detected at 157.60, 151.71 and 161.17. Obviously, the downfield shift in these signals in the ^1^H and ^13^C NMR spectra upon the introduction of the electron-withdrawing substituent (**3c**) is due to the outflow of electron density from this fragment, while in the case of the methoxy-substituent compound **3b**, the opposite effect is observed relative to the unsubstituted derivative **3a**. In the ^15^N NMR spectra, the signals of the nitrogen atoms are also closest to each other in the case of compound **3c** (149.91 and 151.46 ppm), indicating the most effective delocalization of the positive charge. In a similar spectrum of the methoxy-substituted derivative **3b**, the opposite effect is observed, since the positions of these signals are further apart (146.73 and 159.30 ppm) compared to the spectrum of the unsubstituted compound **3a** (149.08 and 159.21 ppm). All these facts indicate that in DMSO-*d*_6_ solutions, the most effective conjugation in the molecule is realized in compound **3c** with a methoxycarbonyl substituent.

In the region of aliphatic protons, in addition to the signals of the CH_3_ groups, the signals of the propanesulfonate fragment are also observed. In the strongest field, the signals of the –CH_2_– group are located, which are detected as multiplet or quartet signals in the region of 2.15–2.17 ppm. In a weaker field, the triplet signals of the –CH_2_–S (2.54, 2.55 and 2.55 ppm) and N^+^–CH_2_– (4.72, 4.76 and 4.73 ppm) groups are observed. As was expected, the substituents in the 5′ position did not affect the positions of these signals. The doublet signals of protons 1′ and 2′ appear at 7.54 and 7.89, 7.60 and 7.90, 7.66 and 7.85 ppm in the spectra of compounds **3a**–**c**, respectively. The values of the *J*-constants (from 16.5 to 17 Hz) indicate their *trans*-configuration.

### 2.2. UV-Vis Spectral Studies

**Figure 1:**
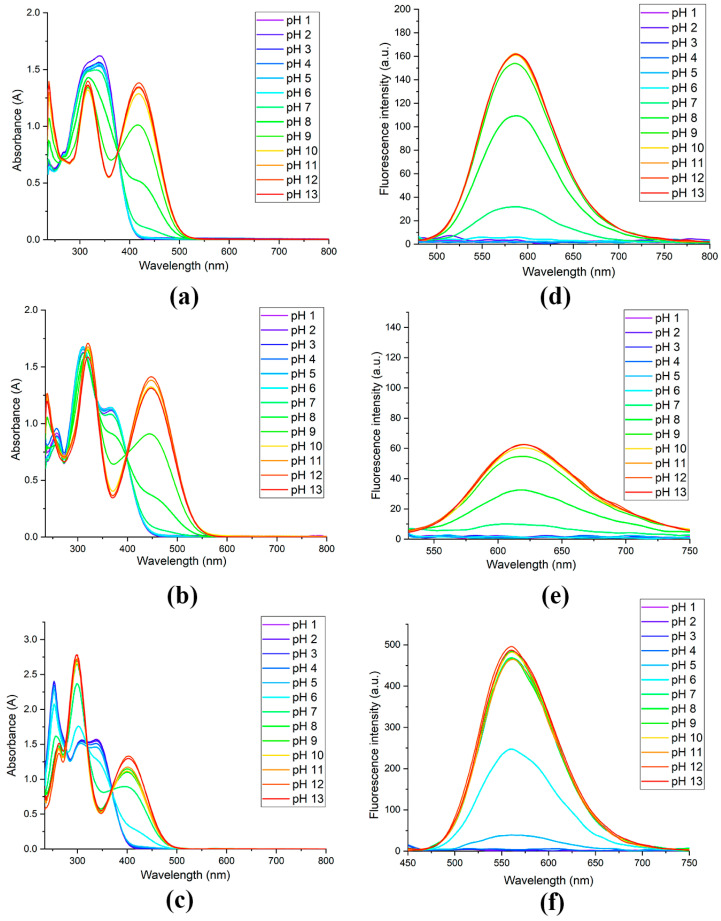
Absorption and photoluminescence spectra (*λ_ex_* = 440 nm) of compounds **3a** (**a**,**d**), **3b** (**b**,**e**) and **3c** (**c**,**f**) (100 μM) in pH buffers.

The spectral and photoluminescence properties of the obtained compounds were studied in phosphate buffer solutions with different pH values (Table 1 and Figure 1). At high acidity values, all compounds are characterized by the absence of fluorescence and three short-wavelength absorption maxima. With an increase in pH value, the intensity of the absorption maxima at 339, 364, and 339 nm for solutions of compounds **3a**–**c**, respectively, gradually decreased, while new absorption maxima appeared at 419, 447, and 402 nm (Figure 1). Apparently, the electron-withdrawing methoxycarbonyl substituent caused a hypsochromic shift in the long-wavelength absorption maximum by 17 nm relative to unsubstituted compound **3a**, and the introduction of an electron-donating substituent into the structure provoked its bathochromic shift by 28 nm.

**Scheme 2:**
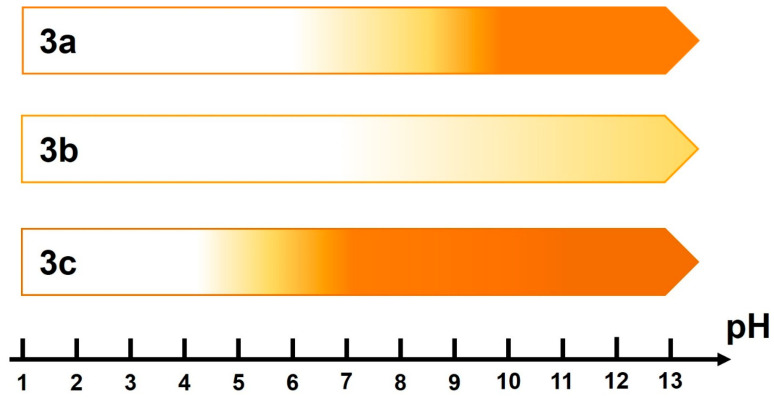
Graphic representation of fluorescence intensity versus pH. The darker the shade of orange, the more intense the fluorescence.

For compounds **3a** and **3b**, the photoluminescence response is observed at a neutral pH, while in the case of dye **3c**, fluorescence begins to appear already at pH = 5. Moreover, the hypsochromic shift in the photoluminescence maximum in the case of compound **3c** relative to the unsubstituted derivative **3a** was 27 nm, while the introduction of the methoxy group caused a bathochromic shift by 34 nm due to its electron-donating effect. Compound **3b** is characterized by a very weak fluorescence response with a quantum yield of 0.001. For unsubstituted compound **3a**, a more noticeable photoluminescence is noted, the intensity of which varies in the pH range of 7–9. However, the quantum yield values at maximum pH still remain low (0.003). The dye **3c**, on the contrary, demonstrates bright fluorescence with a quantum yield of 0.016, and its fluorescence intensity changes in the pH range of 5–7. A graphical representation of the dependence of fluorescence intensity of compounds **3a**–**c** on pH in phosphate buffer solutions is shown in Scheme 2.

**Figure 2:**
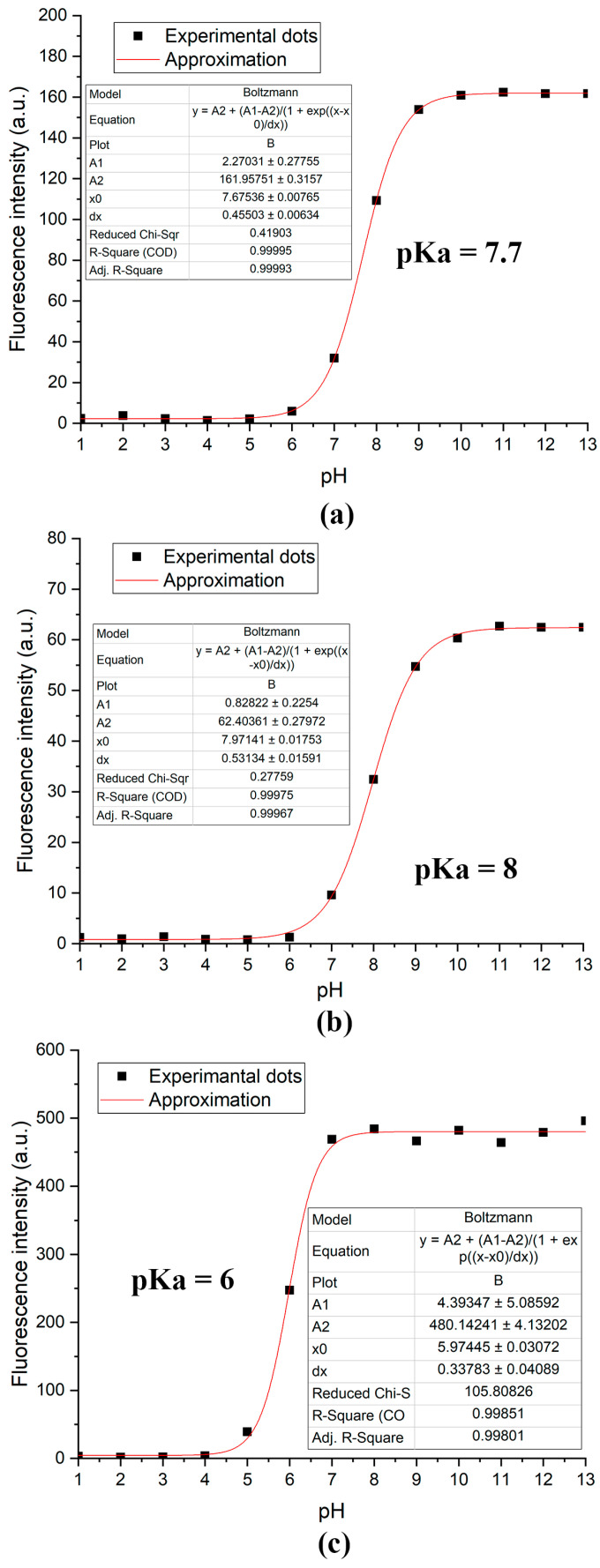
The dependence curve of the fluorescence intensity at the maximum on the pH and pKa value for compounds **3a** (**a**), **3b** (**b**), and **3c** (**c**).

By plotting the fluorescence intensity at its maximum versus pH, we were able to characterize the resulting dyes using pKa values (Figure 2). The dependence of the pKa value on the substituent at position 5′ is the reason for the different ranges of performance of the resulting probes. It should be noted that the introduction of a methoxy group slightly increases the pKa value, while the introduction of a methoxycarbonyl substituent decreases it by 1.7. The value of pKa = 6 indicates that this dye can be used in the pH range of 4.5–7.5, which is well suited for studying bacterial biofilms [27,43]. It is important that, unlike other dyes that function in this pH range [44], compound **3c** was obtained from inexpensive reagents using a simple condensation reaction.

We also investigated the changes in the absorption and photoluminescence spectra of solutions of compounds **3a**–**c** in DMSO upon the addition of hydrochloric acid and DIPEA. Without the addition of bases or acids, compounds **3a** and **3b** did not exhibit photoluminescence. For the latter, no significant changes in the absorption spectra were observed upon the addition of acids and bases, with only a slight decrease in the absorption intensity upon the addition of acid or DIPEA (Figures S27 and S29 in ESI). In the case of compound **3a**, the addition of acid also resulted in an insignificant decrease in the intensity of the absorption maxima (Figure S28 in ESI). However, during titration with DIPEA, the absorption intensity increased more noticeably, and upon the addition of 4 or more molar equivalents, weak fluorescence with a maximum at approximately 615 nm appeared (Figures S25 and S26 in ESI).

**Figure 3:**
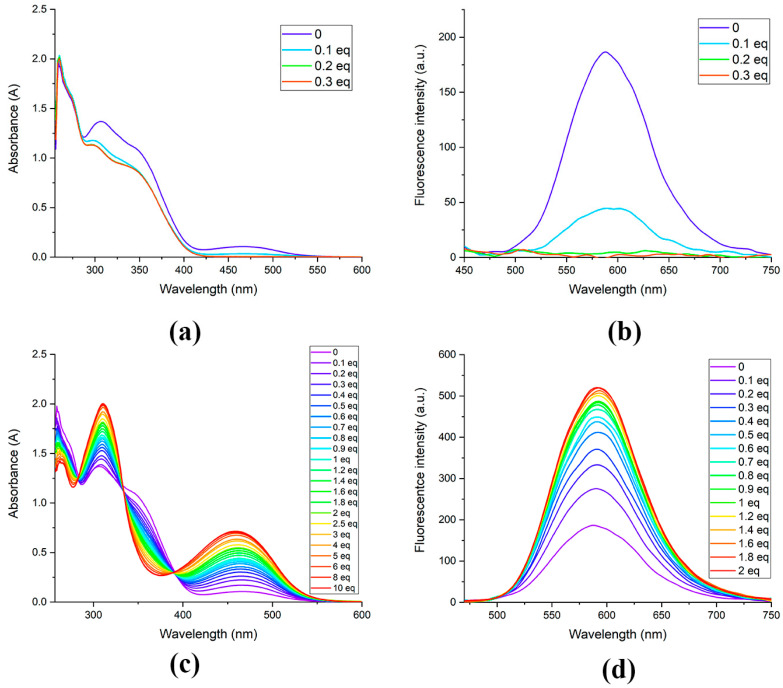
Absorbance (**a**,**c**) and photoluminescence (*λ_ex_* = 440 nm) (**b**,**d**) spectra changes in DMSO solution of compound **3c** (100 μM) during the addition of HCl (**a**,**b**) and DIPEA (**c**,**d**).

Compound **3c** proved to be a much more suitable fluorescent sensor for bases and acids. In the absence of bases and acids, its solution exhibited photoluminescence with a maximum at approximately 591 nm (Figure 3). The addition of just 0.1 molar equivalent of HCl significantly quenched the photoluminescence, while the addition of 0.2 molar equivalents of acid completely eliminated the fluorescence (Figure 3b). Titration with DIPEA resulted in a significant increase in the fluorescent signal, with the maximum intensity increasing by a factor of 2 with the addition of 0.3 molar equivalents, and by a factor of 2.8 with the addition of 1.8 molar equivalents of base (Figure 3d).

**Scheme 3:**
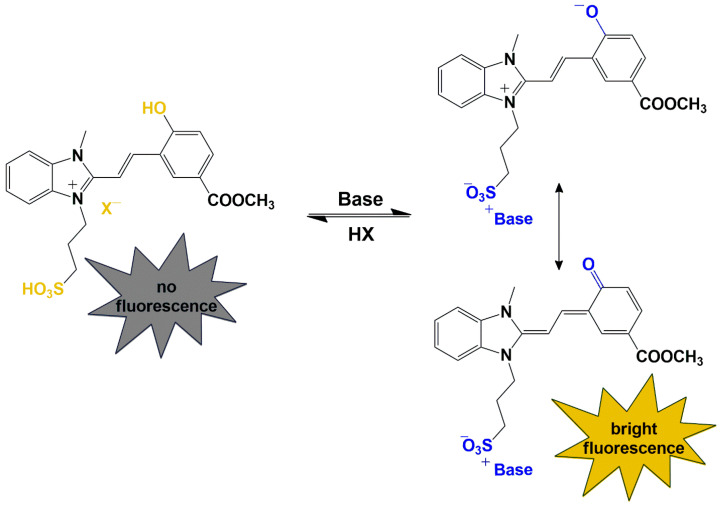
Structural transformations and fluorescent properties changes in benzimidazolium derivatives **3a–c** upon the addition of acids or bases.

Thus, we studied the effect of substituents at position 5′ on the spectral and fluorescence characteristics of benzimidazolium derivatives **3a**–**c**. The methoxycarbonyl substituent provokes a hypsochromic shift in the absorption and fluorescence maxima, while the electron-donating effect of the methoxy substituent leads to the opposite effect. More efficient conjugation in the molecule **3c** leads to the highest fluorescence quantum yields at alkaline pH. The reason for the different values of quantum yields in the series of compounds **3a**–**c** can be explained by the fact that the deprotonated form can be either the betaine or quinoid isomer (Scheme 3). In the case of the methoxycarbonyl substituted derivative **3c**, the efficiency of the conjugation chain increases (as also confirmed by NMR spectroscopy results), and the quinoid isomer most likely predominates, which is the reason for the brighter fluorescence of compound **3c**. Upon addition of acid or in buffer solutions with a pH < 7, protonation of the sulfonate group and charge localization due to salt formation are most likely observed. This transformation leads to a decrease in molecule polarity and the disappearance of fluorescence (Scheme 3). Upon addition of a base, the molecular structure also undergoes some changes. Deprotonation of the hydroxy group occurs, leading to significant polarization of the molecule. This results in the formation of a donor–*π*–acceptor (D–*π*–A) system in the molecule, which enhances fluorescence. When studying the methoxycarbonyl derivative **3c**, this effect is more noticeable due to the more efficient conjugation and the additional acceptor effect of the substituent at position 5 of the molecule.

### 2.3. Biological Studies

**Figure 4:**
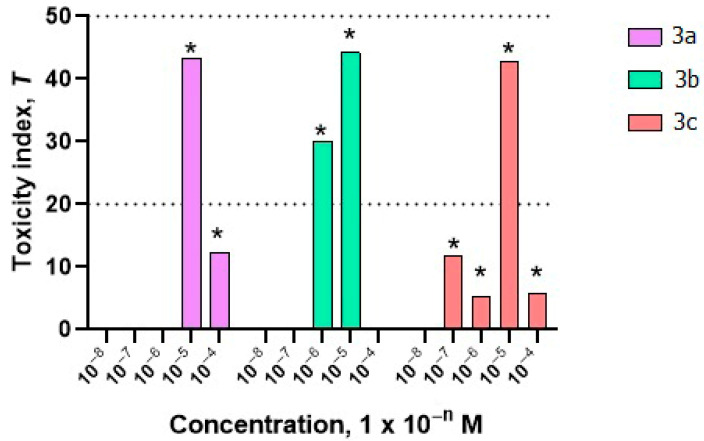
Toxicity of compounds **3a**–**c** at concentrations of 10^−8^–10^−4^ M using the *V. aquamarinus* strain VKPM B-11245. The dotted lines in the diagram indicate the boundaries of the zones of acceptable toxicity, toxicity, and high toxicity. * Differences from the control are statistically significant, *t*-test; *p* < 0.05.

**Figure 5:**
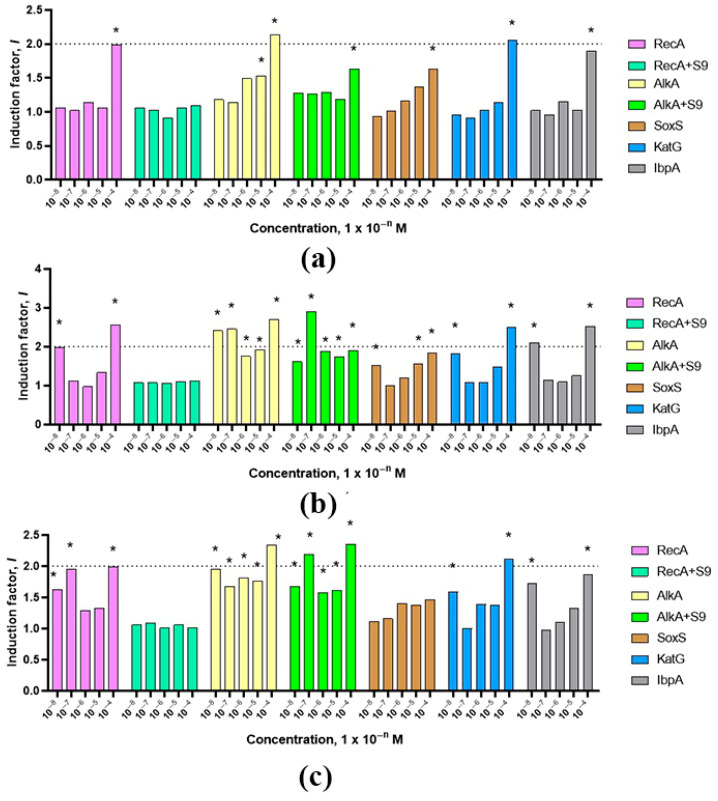
Changes in the toxicity of compounds **3a** (**a**), **3b** (**b**), **3c** (**c**), depending on concentrations (10^−8^–10^−4^ M). The dotted line in the diagram indicates the boundary between weak and moderate toxicity. * Differences from the control are statistically significant, *t*-test; *p* < 0.05.

Whole-cell bacterial lux biosensors were used to study the integral toxicity and mechanisms of cellular toxicity of compounds **3a**–**c**. The results of toxicity bioassays using biosensors are shown in Figure 4 and Figure 5 and in Table S6. Bioassay of integral toxicity showed that all studied compounds are toxic to prokaryotic cells at a concentration of 1 × 10^−5^ M (Figure 4).

Genotoxic effects of moderate strength (activation of the SOS-response) were recorded for all compounds with the *E. coli* MG1655 (pRecA-lux) biosensor at a concentration of 10^−4^ M and characterize the studied substances as direct mutagens. A moderate DNA alkylation effect was also detected in the concentration range of 10^−8^–10^−4^ M for substances **3b** and **3c**. For the substance **3a**, a moderate genotoxic effect was detected only at a concentration of 10^−4^ M. Using the *E. coli* MG1655 (pAlkA-lux) biosensor, weak promutagenic effects were also recorded, with the exception of substances **3b** and **3c** at a concentration of 10^−7^, for which a moderate promutagenic effect was recorded (Figure 5).

All three compounds studied exhibited mild toxic effects associated with superoxide oxidative stress. In the case of peroxide oxidative stress, compounds **3a**–**c** also exhibited mild toxic effects, with the exception of a concentration of 10^−4^ M, at which the compounds exhibited moderate toxicity.

Compound **3b** at concentrations of 10^−8^ and 10^−4^ M exhibited a moderate effect on protein damage. Compounds **3a** and **3b** at all concentrations studied and compound **3b** at concentrations ranging from 10^−7^ to 10^−5^ M exhibited a weak toxic effect on cellular proteins.

**Figure 6:**
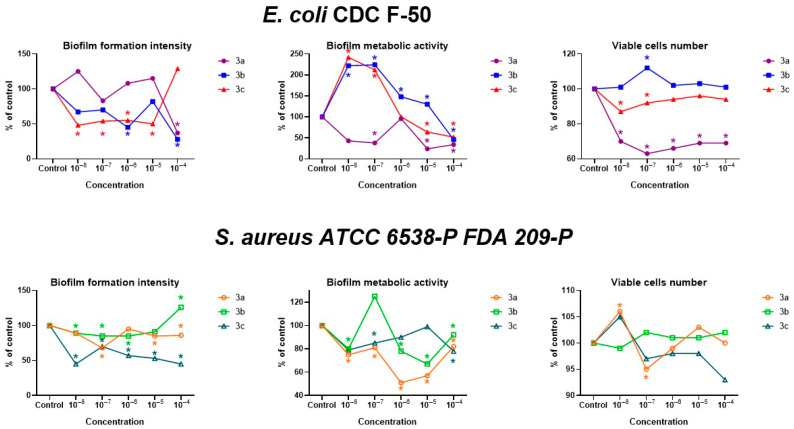
The effect of compounds **3a**–**c** on the biomass, metabolic activity, and viable cell count of bacterial biofilms of *E. coli* CDC F-50 and *S. aureus* ATCC 6538-P FDA 209-P strains, depending on concentration (10^−8^–10^−4^ M). * Differences from control are statistically significant, *t*-test; *p* < 0.05.

Figure 6 and Tables S2–S6 (in ESI) present data on the effect of the studied substances on the biofilm biomass, the number of viable cells, and the metabolic activity of bacterial cells of the *E. coli* CDC F-50 and *S. aureus* ATCC 6538-P FDA 209-P strains.

Substance **3a** exerted a suppressive effect on all the studied parameters in the *E. coli* CDC F-50 strain. The metabolic activity of cells was particularly significantly reduced at concentrations of 10^−5^ and 10^−4^ M. Substance **3b** exerted a suppressive effect on biofilm biomass at concentrations of 10^−6^ and 10^−4^ M and also reduced metabolic activity at a concentration of 10^−4^ M. Other concentrations caused an increase in metabolic activity to 130–224%, and the number of viable cells also increased at a concentration of 10^−7^ M. Compound **3c** had a suppressive effect on biofilm biomass at concentrations of 10^−5^–10^−8^ M. Concentrations of 10^−7^ and 10^−8^ M inhibited the number of viable cells, but these same concentrations increased metabolic activity by 212 and 242%, respectively. Concentrations of 10^−5^ M and 10^−4^ M inhibited the metabolic activity of *E. coli* CDC F-50 cells by almost 2-fold.

In relation to *S. aureus* ATCC 6538-P FDA 209-P, substance **3a** also suppressed biofilm biomass and metabolic activity at all concentrations studied. The number of viable cells slightly increased at a concentration of 10^−8^ M (106% of the control) and decreased at a concentration of 10^−7^ M (95% of the control). Compound **3b** did not affect the number of viable cells, but concentrations of 10^−8^–10^−6^ M suppressed biofilm biomass, while 10^−4^ M stimulated it. Metabolic activity was suppressed at all concentrations. Metoxycarbonyl-substitued derivative **3c** also reduced biofilm biomass at all concentrations to 70–45% of the control, did not affect viable cells, and reduced metabolic activity (at concentrations of 10^−8^, 10^−7^, and 10^−4^ M).

Thus, the studied substances have a predominantly suppressive effect on biofilm biomass, the number of living cells, and their metabolic activity in *E. coli* CDC F-50 and *S. aureus* ATCC 6538-P FDA 209-P.

### 2.4. Fluorescent Microscopy

Based on spectral and biological studies, we selected dye **3c** as the most promising substance for use as a fluorescent probe. The first bioimaging experiment was carried out using *E. coli* CDC F-50 biofilm treated with the dye solution for 3 h. Fluorescence was excited by a laser with a wavelength of 458 nm or in two-photon mode with a wavelength of 725 nm (Figures S31, S32, S35 and S36 in ESI). It should be noted that the possibility of using excitation wavelengths in the biological window range (650–1000 nm) is particularly relevant, as such radiation is the least destructive and can penetrate sufficiently deep into a living organism due to minimal scattering and absorption by biological objects.

**Figure 7:**
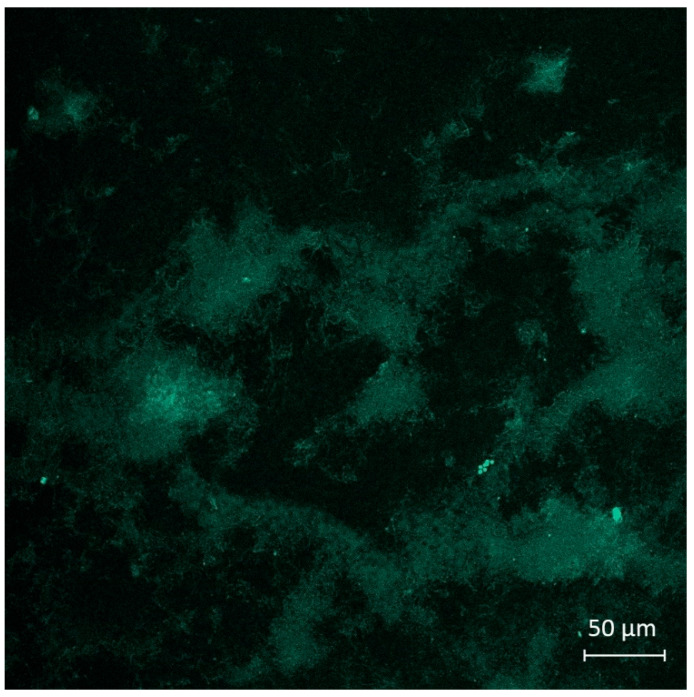
The image of *E. coli* CDC-F50 biofilm treated by a solution of dye **3c** (10^−4^ M, excitation in multiphoton mode with *λ* = 725 nm) during 3 h, obtained as a result of the bioimaging experiment.

Based on the obtained results, it can be assumed that after 3 h, the substance stained only the extracellular matrix of the bacterial biofilm (Figure 7), and the fluorescence spectrum (Figures S35 and S36 in ESI) almost completely coincided with that obtained during spectral studies in phosphate buffer solutions with a pH > 5.

**Figure 8:**
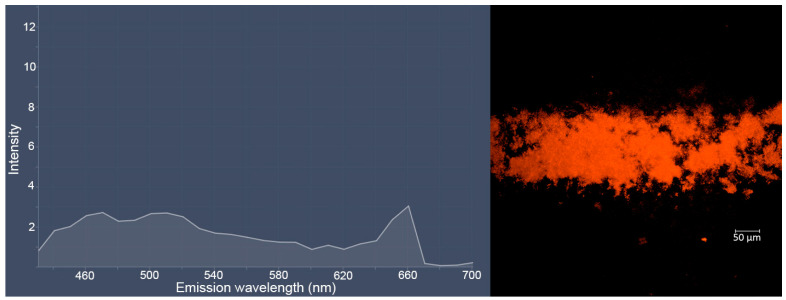
Lambda-stack (**left**) and snap (**right**) images of *E. coli* CDC-F50 biofilm treated by a solution of dye **3c** (10^−4^ M, excitation in multiphoton mode with *λ* = 725 nm) during 24 h obtained as a result of the bioimaging experiment.

**Figure 9:**
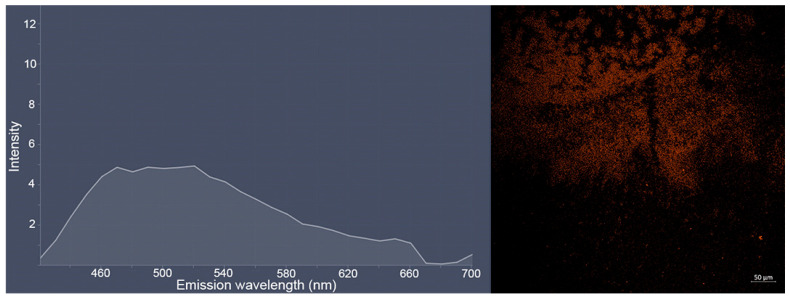
Lambda-stack (**left**) and snap (**right**) images of *S. aureus* ATCC 6538-P FDA 209-P biofilm treated by solution of dye **3c** (10^−4^ M, excitation in multiphoton mode with *λ* = 725 nm) during 24 h obtained as a result of the bioimaging experiment.

With longer staining (24 h) of bacterial *E. coli* CDC-F50 and *S. aureus* ATCC 6538-P FDA 209-P biofilms, the clusters of individual bacterial cells become clearly visible, which most likely indicates penetration of dye **3c** into the cells (Figure S37 in ESI). The obtained images of biofilms and their emission spectra are given below (Figure 8 and Figure 9). Interestingly, in the case of *E. coli*, a new photoluminescence maximum appears in the region of 660 nm.

Thus, the obtained and studied dye **3c** can be used to visualize both the extracellular matrix and bacterial biofilm cells when excited by a laser at a wavelength of 458 nm or in two-photon mode using excitation with a wavelength of 725 nm. The sensitivity of the fluorescent response to pH values makes it a promising non-invasive probe for studying the acidity of bacterial biofilms [27] in the biological pH range (4.5–7.5).

### 2.5. Molecular Docking

Computer modeling using the molecular docking method was performed for a series of benzimidazolium derivatives **3a**–**c** to assess their potential ability to bind to DNA and determine the most preferred interaction mode (Table 2).

In all cases, binding in the minor groove of DNA turned out to be the most energetically favorable. For the unsubstituted derivative **3a**, the calculated free energy of binding (*∆G*) was −7.75 kcal/mol, where the inhibition constant (*K_i_*) is 2.09 μM. Analysis of the contributions of individual interactions showed that the binding is primarily due to significant Van der Waals contacts (*E_vdWHD_* = −9.09 kcal/mol), indicating good steric complementarity of the molecule to the shape of the minor groove. The introduction of a methoxy substituent led to increased binding in the minor groove (*∆G* = −8.07 kcal/mol, *K_i_* = 1.21 μM). The improvement in affinity is associated with an increase in the contribution of electrostatic interactions (*E_el_* = −1.02 versus −0.74 kcal/mol for **3a**), which suggests the formation of an additional hydrogen bond between the atoms of the methoxy group and DNA. The most significant interactions with DNA can be expected for the derivative with a metoxycarbonyl substituent **3c** (*K_i_* = 637.36 nM, *∆G* = −8.45 kcal/mol). Notably, the sharp increase in affinity compared to the methoxy derivative is due not to electrostatics (*E_el_* = −0.91 kcal/mol) but to exceptionally strong van der Waals interactions (*E_vdWHD_* = −10.23 kcal/mol).

Thus, the molecular docking results allow us to conclude that all studied benzimidazolium derivatives, **3a**–**c,** exhibit the characteristics of potential selective ligands for the DNA minor groove. Importantly, in this part of the study, dye **3c** was also preferable among other molecules and demonstrated the highest predicted binding affinity.

## 3. Experimental

### 3.1. Materials and Methods

All reagents were purchased from Acros Organics (Geel, Belgium), Alfa Aesar (Ward Hill, MA, USA), Merck (Darmstadt, Germany), and TCI (Shanghai, China) and were used without further purification. The following reagents were used in the study: *o*-phenylenediamine (98% purity, Acros Organics, Geel, Belgium), 1,3-propanesultone (99% purity, TCI, Shanghai, China), salicylic aldehyde (99% purity, Alfa-Aesar, Ward Hill, MA, USA) 2-Hydroxy-5-methoxybenzaldehyde (98% purity, Merck, Darmstadt, Germany), and Methyl 3-formyl-4-hydroxybenzoate (97% purity, Merck, Darmstadt, Germany). Organic solvents were purified and dried according to standard procedures.

NMR ^1^H, ^13^C, and ^15^N spectra were recorded on a Bruker AVANCE-600 (Billerica, MA, USA) spectrometer (600 MHz for ^1^H, 151 MHz for ^13^C, 60 MHz for ^15^N) at the Collective Use Center “Molecular Spectroscopy” of the Southern Federal University. Chemical shifts (*δ*) are reported in ppm relative to the residual solvent signals of DMSO-*d*_6_ (δ_H_ 2.49 ppm, δ_C_ 39.50 ppm). For ^15^N spectra, shifts are referenced to nitromethane (δ_N_ 384 ppm). FT-IR spectra were acquired using a Jasco FT/IR-6800R spectrometer (Tokyo, Japan) equipped with an ATR PRO ONE accessory for Attenuated Total Reflectance (ATR) measurements on solid samples. High-Resolution Mass Spectra (HRMS) were obtained on a Bruker UHR-TOF Maxis™ Impact mass spectrometer (Billerica, MA, USA) using Electrospray Ionization (ESI). Melting points were determined using a Fisher–Johns apparatus (Thermo Fisher Scientific, Waltham, MA, USA).

The UV-Vis spectra were recorded on an Agilent Cary 60 spectrophotometer. Emission spectra were registered on an Agilent Cary Eclipse fluorescence spectrophotometer (Santa Clara, CA, USA). Stock solutions of compounds **3**, diisopropylethylamine (DIPEA), and HCl (1.00 × 10^−2^ M) in DMSO were prepared immediately before measurements by dissolving the exact sample and diluting to the required volume. Pure fluorescein (97.6% purity, Acros Organics, Geel, Belgium) was used to determine the quantum yield.

### 3.2. Synthesis

**3-[2-[(E)-2-(2-Hydroxyphenyl)vinyl]-3-methylbenzimidazol-1-ium-1-yl]propane-1-sulfonate 3a.** A portion of 3-(2,3-dimethylbenzimidazol-1-ium-1-yl)propane-1-sulfonate **1** (0.536 g, 2 mmol) was added to a vigorously stirred, boiling solution of salicylic aldehyde **2a** (0.244 g, 2 mmol, 0.22 mL) in methanol (10 mL). Piperidine (0.05 mL) was added dropwise over 5 min. The resulting mixture was refluxed under argon for 12 h. The reaction progress was monitored using thin-layer chromatography. After cooling to room temperature, the solvent was concentrated to ~5 mL under reduced pressure. The orange precipitate was collected by filtration, then washed with cold ethanol (3 × 2 mL) and dried in vacuo. Yield 0.380 g, 51.1%. mp = 303 °C. **HRMS (ESI):** *m*/*z* calcd for C_19_H_20_N_2_NaO_4_S [M + Na]^+^: 395.1036; found: 395.1034. **IR (cm^−1^):** 3038 (O–H), 2935, 2881 (C–H_alk_), 1625, 1599, 1579 (C=Cₐᵣ), 1519 (C=N^+^), 1224 (Cₐᵣ–N), 1192 (C–O), 1151, 1110 (S=O), 1027 (C–N), 745 (C–S), 726 (S–O). **^1^H NMR**: δ = 2.11–2.20 (m, 2H, –CH_2_–), 2.54 (t, *J* = 6.8 Hz, 2H, –CH_2_–S), 4.07 (s, 3H, N^+^–CH_3_), 4.72 (t, *J* = 7.5 Hz, 2H, N^+^–CH_2_–), 6.90 (t, *J* = 7.5 Hz, 1H, H-4′), 6.96 (d, *J* = 8.2 Hz, 1H, H-6′), 7.30 (td, *J* = 7.2, 1.2 Hz, 1H, H-5′), 7.54 (d, *J* = 16.8 Hz, 1H, H-1′), 7.60–7.67 (m, 2H, H-5, H-6), 7.89 (d, *J* = 16.5 Hz, 1H, H-2′), 7.91 (d, *J* = 8.1 Hz, 1H, H-3′), 7.96–8.02 (m, 1H, H-4), 8.06–8.12 (m, 1H, H-7), 10.46 (s, 1H, –OH). **^13^C NMR:** δ = 25.89 (–CH_2_–), 33.70 (N^+^–CH_3_), 44.86 (N^+^–CH_2_–), 48.04 (–CH_2_–S), 107.37 (C-1′), 113.46 (C-7), 113.50 (C-4), 116.89 (C-6′), 120.00 (C-4′), 121.68 (C-7′), 126.73 (C-5′), 126.83 (C-6), 129.66 (C-2′), 131.51 (C-8), 132.83 (C-5), 132.99 (C-9), 142.66 (C-3′), 149.02 (C-2), 157.60 (C–OH). **^15^N NMR:** δ = 149.08 (N^+^–CH_3_), 159.21 (N^+^–CH_2_–).

**3-[2-[(E)-2-(2-Hydroxy-5-methoxyphenyl)vinyl]-3-methylbenzimidazol-1-ium-1-yl]propane-1-sulfonate 3b.** The synthesis from 2-hydroxy-5-methoxybenzaldehyde **2b** and 3-(2,3-dimethylbenzimidazol-1-ium-1-yl)propane-1-sulfonate **1**, along with product isolation, was carried out analogously to compound **3a**. Yield 0.350 g (43.5%). mp = 333 °C. **HRMS (ESI):** *m*/*z* calcd for C_20_H_22_N_2_NaO_5_S [M + Na]^+^: 425.1142; found: 425.1141. **IR (cm^−1^):** 3091 (O–H); 3029, 2963 (C–H_alk_); 2921 (C_ar_–O–CH_3_); 1626, 1613 (C=C_ar_); 1530 (C=N^+^); 1274, 1212 (C_ar_–N); 1196 (C–O); 1169 (C_ar_–O–C); 1150 (S=O); 1031 (C–N); 767 (C–S); 720 (S–O). **^1^H NMR:** δ = 2.16 (q, *J* = 7.0 Hz, 2H, –CH_2_–), 2.55 (t, *J* = 6.6 Hz, 2H, –CH_2_–S), 3.77 (s, 3H, O–CH_3_), 4.08 (s, 3H, N^+^–CH_3_), 4.76 (t, *J* = 7.5 Hz, 2H, N^+^–CH_2_–), 6.87 (d, *J* = 8.9 Hz, 1H, H-3′), 6.92 (dd, *J* = 8.9, 3.0 Hz, 1H, H-4′), 7.54 (d, *J* = 3.0 Hz, 1H, H-6′), 7.60 (d, *J* = 16.8 Hz, 1H, H-1′), 7.62–7.65 (m, 2H, H-5, H-6), 7.90 (d, *J* = 16.8 Hz, 1H, H-2′), 7.96–8.00 (m, 1H, H-4), 8.05–8.10 (m, 1H, H-7), 9.97 (s, 1H, –OH). **^13^C NMR:** δ = 25.89 (–CH_2_–), 33.79 (N^+^–CH_3_), 44.86 (N^+^–CH_2_–), 47.85 (–CH_2_–S), 56.39 (O–CH_3_), 107.44 (C-1′), 112.40 (C-6′), 113.42 (C-7), 113.49 (C-4), 117.86 (C-3′), 120.39 (C-4′), 121.81 (C-7′), 126.72 (C-5), 126.82 (C-6), 131.46 (C-8), 132.93 (C-9), 142.30 (C-2′), 148.97 (C-2), 151.71 (C–OH), 153.02 (C-5′). **^15^N NMR:** δ = 146.73 (N^+^–CH_3_), 159.30 (–CH_2_–N^+^).

**3-[2-[(E)-2-(2-Hydroxy-5-(methoxycarbonyl)phenyl)vinyl]-3-methylbenzimidazol-1-ium-1-yl]propane-1-sulfonate 3c.** The synthesis from 5-methoxycarbonyl-salicylaldehyde **2c** and 3-(2,3-dimethylbenzimidazol-1-ium-1-yl)propane-1-sulfonate **1**, along with product isolation, was carried out analogously to compound **3a.** Yield: 0.666 g (77.4%). mp = 315 °C. **HRMS (ESI):** *m*/*z* calcd for C_21_H_22_N_2_NaO_6_S [M + Na]^+^: 453.1091; found: 453.1088. **IR (cm^−1^):** 3469 (O–H); 3075, 3038 (C–H_alk_); 2952 (C–O–CH_3_); 1701 (C=O); 1679 (C(=O)–O); 1626, 1602 (C=C_ar_); 1503 (C=N^+^); 1244, 1210 (C_ar_–N); 1196 (C–O); 1175 (C–O–C); 1166, 1123 (S=O); 1035 (C–N); 756 (C–S); 728 (S–O). **^1^H NMR:** δ = 2.17 (q, *J* = 7.2 Hz, 2H, –CH_2_–), 2.55 (t, *J* = 7.1 Hz, 2H, –CH_2_–S), 3.84 (s, 3H, O–CH_3_), 4.10 (s, 3H, N^+^–CH_3_), 4.73 (t, *J* = 7.5 Hz, 2H, N^+^–CH_2_–), 7.08 (d, *J* = 8.6 Hz, 1H, H-3′), 7.66 (d, *J* = 17.0 Hz, 1H, H-1′), 7.67 (dt, *J* = 9.3, 4.0 Hz, 2H, H-5, H-6), 7.85 (d, *J* = 16.8 Hz, 1H, H-2′), 7.90 (dd, *J* = 8.6, 2.2 Hz, 1H, H-4′), 8.02 (td, *J* = 7.2, 3.6 Hz, 1H, H-4), 8.13 (td, *J* = 7.2, 3.6 Hz, 1H, H-7), 8.42 (d, *J* = 2.2 Hz, 1H, H-6′), 11.55 (s, 1H, –OH). **^13^C NMR:** δ = 25.36 (–CH_2_–), 33.08 (N^+^–CH_3_), 44.50 (N^+^–CH_2_–), 47.64 (–CH_2_–S), 51.78 (O–CH_3_), 108.68 (C-1′), 113.04 (C-4, C-7), 116.49 (C-3′), 120.84 (C-5′), 121.15 (C-7′), 126.27 (C-5), 126.38 (C-6), 131.00 (C-8a), 131.47 (C-6′), 132.19 (C-9a), 133.07 (C-4′), 141.64 (C-2′), 148.36 (C-2), 161.17 (C–OH), 165.66 (C(=O)–O). **^15^N NMR:** δ = 149.91 (N^+^–CH_3_), 151.46 (N^+^–CH_2_–).

### 3.3. Biological Studies

Strains *Staphylococcus aureus* ATCC 6538-P FDA 209-P and *Escherichia coli* CDC F-50 served as a model object for studying bacterial biofilms.

The following whole-cell bioluminescent bacterial sensors were used to evaluate the toxicity of substances: *E. coli* MG1655 (pRecA-lux), *E. coli* MG1655 (pColD-lux), *E. coli* MG1655 (pAlkA-lux), *E. coli* MG1655 (pIbpA-lux), *E. coli* MG1655 (pXen7), and *Vibrio aquamarinus* VKPM B-11245.

The test compounds were dissolved in DMSO to a concentration of 1 × 10^−2^ M. Then they were diluted with a water–DMSO mixture to a concentration of 1 × 10^−4^ M. Further dilutions were prepared by adding water. The control solutions corresponded to the solvent at the investigated concentration of the substance. A more detailed description of biotesting methods is given in the ESI [53,54,55,56,57,58,59,60,61,62,63,64].

### 3.4. Molecular Docking

To ensure rigorous comparability of the results, molecular docking was performed following an established protocol previously used for investigating cyanine dye-DNA interactions [65]. All computational docking procedures were executed using AMDock 1.5.2 (Linux version) [66] with the AutoDock 4.2.6 engine [67]. The canonical B-DNA structure (PDB ID: 1BNA [68]) served as the receptor model. Preliminary structure preparation conducted in AMDock included removal of crystallographic water molecules and addition of all polar hydrogen atoms. The docking grid was configured with the following parameters: center coordinates (x = 15, y = 20, z = 10) and grid dimensions (Δx = 25, Δy = 25, Δz = 40) Å. Result visualization and structural analysis were performed using the PyMOL 3.1 Molecular Graphics System [69].

A search for optimized structures for molecular docking studies was performed using Orca 6.0 software [70,71] in the scope of DFT approach in the PBE0 function [72] with the def2-TZVP [73] basis set, taking into account the atom-pairwise dispersion correction with the Beck–Johnson damping scheme (D3BJ) [74,75]. All simulations were carried out using CPCM solvent model [76] to account for the effects of water solution. Coordinates of optimized structures are given in the ESI (Tables S7–S9).

### 3.5. Fluorescent Microscopy

The images of bacteria *S. aureus* ATCC 6538-P FDA 209-P and *E. coli* CDC F-50 were obtained with laser scanning microscope LSM 880 (Carl Zeiss Microscopy, Leipzig, Germany) equipped with a 20 × objective (W Plan-Apochromat 20×/1.0 DIC D = 0.17 M27 VIS-IR). The imaging was performed in confocal mode. Dye **3c** was excited using an argon laser (Ar 458) at a wavelength of 458 nm. The emission light was collected in the range of 550–610 nm using a multichannel gallium arsenide phosphid (GaAsP) photomultiplier tube (Carl Zeiss Microscopy, Leipzig, Germany).

In the Lambda Mode, a series of images (λ-stack) with a discrete light bandwidth was obtained using a 34-channel detector in the LSM 880. In our experiments, the detector covers a spectral width of approximately 380–700 nm with a step ≈10 nm. Image processing and analysis were carried out using ZEN Black Edition 2.3. and ZEN Blue Edition 2.6 software.

Two-photon imaging was performed using a Chameleon Discovery femtosecond laser (Coherent, Glasgow, UK) integrated with LSM 880, tunable in the range 680–1300 nm, with a short pulse duration (100 fs). It was experimentally established that the excitation of the dye is maximum at a wavelength of 725 nm. The emission light was collected using NDD detector (Carl Zeiss Microscopy, Leipzig, Germany) based on gallium phosphide arsenide in the range of 530–690 nm. The laser power was regulated to minimize photobleaching of the dye and damage to the biological objects.

## 4. Conclusions

Thus, we obtained and studied a series of benzimidazolium derivatives with a propanesulfonate moiety, condensed with salicylic aldehyde and its derivatives. The study of the absorption and photoluminescence properties of compounds in phosphate buffers and DMSO solution, followed by titration with DIPEA or HCl, revealed that the methoxycarbonyl-substituted derivative is the most suitable for use as a fluorescent probe. This may be due to more efficient conjugation and partial charge delocalization within the molecule.

Toxicity of all the obtained compounds was studied using bacterial biofilms and lux-biosensors. Substances exhibit predominantly suppressive effects on biofilm biomass, the number of living cells, and their metabolic activity in *E. coli* CDC F-50 and *S. aureus* ATCC 6538-P FDA 209-P strains.

Bioimaging experiments have shown that dye **3c** can be used to visualize both the extracellular matrix and bacterial biofilm cells when excited by a laser with a wavelength of 458 nm or in multiphoton mode using radiation with a wavelength of 725 nm. Moreover, when penetrating bacteria, it is capable of binding to intracellular objects, such as DNA. This fact is indirectly confirmed by the results of molecular docking.

The combination of the obtained results makes the obtained benzimidazolium derivatives a powerful tool for the ratiometric analysis of acidity in biological systems including bacterial biofilms in the pH range 4.5–7.5 (pKa = 6), opening up broad prospects for their application in fundamental and diagnostic biomedical research.

## Figures and Tables

**Table 1:** Spectral and photoluminescent properties of compounds **3a**–**c** (100 μM) in pH buffers at 293K.

No.	Structure	pH	Absorption, *λ*_max_, nm (ε·10^−4^, M^−1^·cm^−1^)	*λ*^Fl^_max_, nm	Φ_Fl_^a^
**3a**	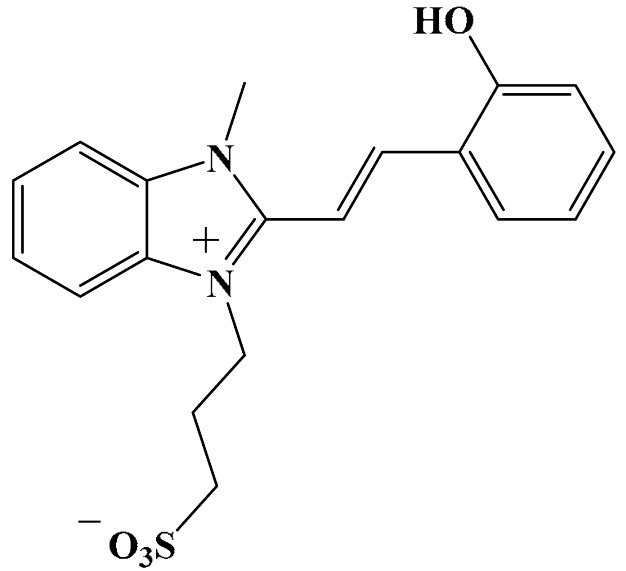	1	239 (0.66), 310 (1.49), 339 (1.56)	–	–
		7	239, 310, 339	587	–
		13	238 (1.36), 316 (1.36), 419 (1.35)	587	0.003
**3b**	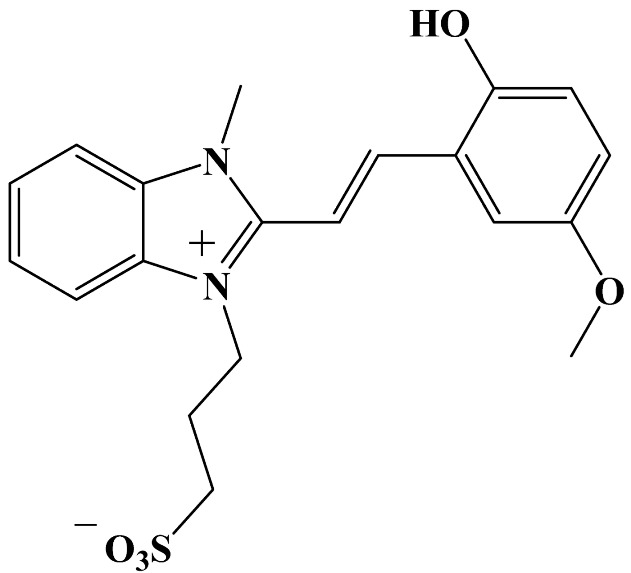	1	258 (0.92), 310 (1.68), 364 (1.14)	–	
		7	258, 312, 364	621	–
		13	239 (1.20), 321 (1.59), 447 (1.31)	621	0.001
**3c**	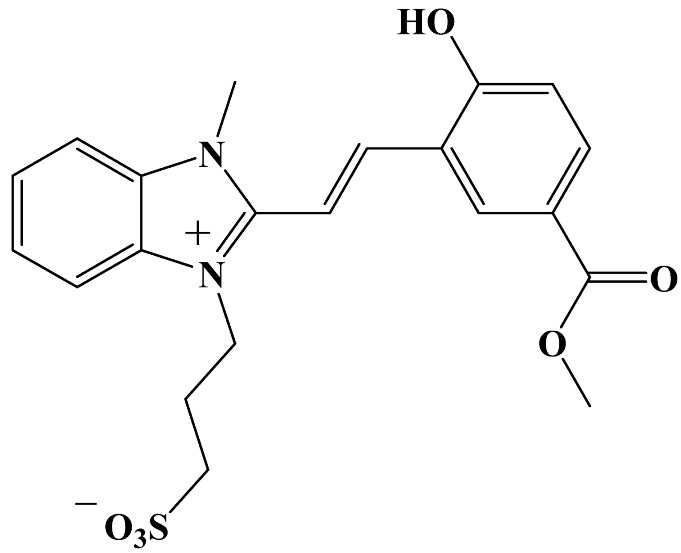	1	253 (2.38), 310 (1.56), 339 (1.53)	–	–
		7	257, 299, 395	560	–
		13	263 (1.51), 298 (2.78), 402 (1.30)	560	0.016

^a^ Quantum yield of fluorescence was estimated using fluorescein in 0.1 M NaOH at 470 nm excitation.

**Table 2:** Results of molecular docking for compounds **3a**–**c** with a DNA molecule (1BNA).

Binding Mode	∆*G_est_*	*E_intmol_*	*E_vdWHD_*	*E_el_*	*E_tint_*	*E_tor_*	*K_i_*
	k_cal_·mol^−1^	k_cal_·mol^−1^	k_cal_·mol^−1^	k_cal_·mol^−1^	k_cal_·mol^−1^	k_cal_·mol^−1^	μM
**3a**							
minor groove	−7.75	−9.84	−9.09	−0.74	−0.86	+2.09	2.09
major groove	−6.84	−8.93	−7.87	−1.06	−0.76	+2.09	9.63
intercalation	−4.38	−6.47	−4.59	−1.88	−2.54	+2.09	617.73
**3b**							
minor groove	−8.07	−10.46	−9.44	−1.02	−0.85	+2.39	1.21
major groove	−6.56	−8.95	−7.59	−1.36	−0.62	+2.39	15.52
intercalation	−4.17	−6.56	−5.65	−0.90	−2.53	+2.39	878.52
**3c**							
minor groove	−8.45	−11.14	−10.23	−0.91	−1.03	+2.68	0.637
major groove	−6.89	−9.57	−8.43	−1.14	−1.60	+2.68	8.97
intercalation	−4.94	−7.62	−6.42	−1.20	−0.97	+2.68	241.20

## Data Availability

The original contributions presented in this study are included in the article/Supplementary Material. Further inquiries can be directed to the corresponding author.

## Supplementary Materials

The following supporting information can be downloaded at: https://www.mdpi.com/article/10.3390/molecules30234622/s1. References [53,54,55,56,57,58,59,60,61,62,63,64] are cited in the Supplementary Materials.
