# Supplementary material for: New Benzimidazole-Based pH-Sensitive Fluorescent Probes
Source: Molecules. 2025 Dec 1;30(23):4622. doi: 10.3390/molecules30234622 (PMC12692881; doi:10.3390/molecules30234622)
Supplement: Supplementary file 1 [file molecules-30-04622-s001.zip › molecules-3965163-supplementary.pdf]

Supplementary Information

New benzimidazole-based pH-sensitive fluorescent probes

Artem D. Pugachev[a*], Ivan N. Bardasov[b], Shorena K. Karchava[c], Tatiana N. Azhogina[c], Maria V. Klimova[c], Alexey E. Matukhno[d], Vitaly S. Dmitriev[a], Gennady S. Borodkin[a], Olga D. Lanovaya[a], Diana Yu. Pobedinskaya[e], Angelina E. Polinichenko[a], Ludmila E. Khmelevtsova[c], Ivan S. Sazykin[c], Marina A. Sazykina[c], Ilya V. Ozhogin[a]

[a] *Institute of Physical and Organic Chemistry, Southern Federal University, 344090 Stachki prosp., 194/2, Rostov-on-Don, Russian Federation*

[b] *Chuvash State University named after I.N. Ulyanov, 428015 Moscow prosp., 15, Cheboksary, Chuvash Republic, Russian Federation*

[c] *Academy of Biology and Biotechnologies, Southern Federal University, 344090 Stachki prosp., 194/1, Rostov-on-Don, Russian Federation*

[d] *Research Center for Neurotechnology, Southern Federal University, 344090 194 Stachka ave., Rostov-on-Don, Russian Federation*

[e] *Department of Chemistry, North-Caucasus Federal University 355017, Stavropol, Pushkin Street, 1, Russian Federation*

**Table of Contents**	**Page No.**
1. Synthesis of intermediate compounds	**S3-S4**
2. NMR and HRMS spectra	**S5-S16**
3. Spectral studies	**S17-S19**
4. Biological studies	**S20-S25**
5. Molecular docking	**S26-S30**
6. Fluorescent microscopy	**S31-S34**

1.Synthesis of intermediate compounds

To obtain targeted compounds, we synthesized the initial derivatives of benzimidazole with a methyl and propanesulfonate group bonded with nitrogen atoms. At the first stage, 2-methylbenzimidazole was synthesized according to the standard technique by heating of the ortho-phenilendiamine with acetic acid in a water bath (yield 46 %, **Scheme S1**). Then it was methylated with methyl iodide in acetone using chopped KOH at room temperature (yield 62.1 %, **Scheme S2**).

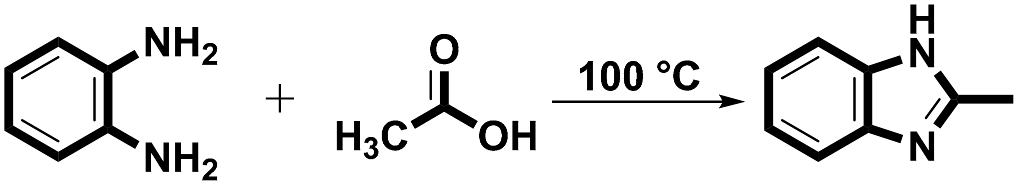

Scheme S1. Synthesis of 2-methylbenzimidazole

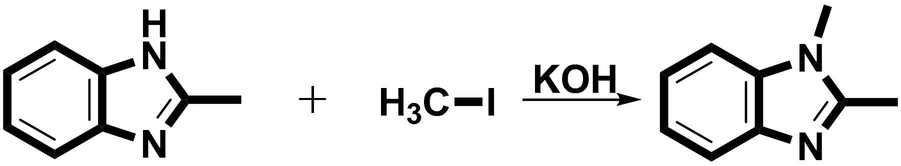

Scheme S2. Synthesis of 1,2-dimethylbenzimidazole.

Propanesulfonate derivatives of benzimidazole were prepared by the action of 1,3-propanesultone (1.5 equiv.) on the 1,2-dimethylbenzimidazole in acetonitrile under argon atmosphere (**Scheme S3**). The yield of the target product was 90.2 %.

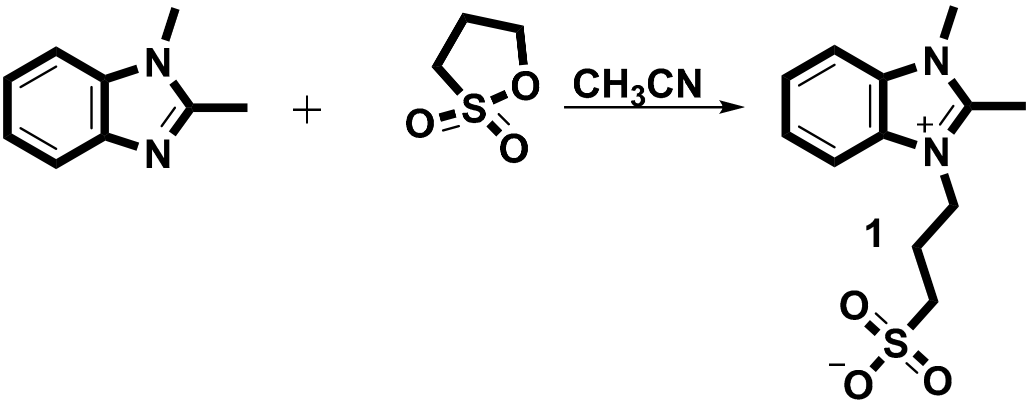

Scheme 3. Synthesis of Sulfopropylbenzimidazolium Derivatives.

3-(2,3-dimethylbenzimidazol-1-yl)propane-1-sulfonate 1. In a 50 ml flask, 0.4392 g (3 mmol) of 2,3-dimethylbenzimidazole was dissolved in 5 ml of acetonitrile and a 1.5-fold excess (0.5496 g, 0.4 ml, 4.5 mmol) of 1,3-propanesultone was added. The reaction mixture was boiled and stirred under argon for 3 hours. Then it was cooled and the resulting white precipitate was filtered and washed with cold acetonitrile and diethyl ether. Yield 0.725 g

(90.2 %). mp = 293 °C. **NMR ^{1}H (DMSO-*d_6*) δ, ppm (*J*, Hz):** 2.09 (q, *J* = 7.2, 2H, $–CH_2–$), 2.50 – 2.55 (m, 2H, $–CH_2–S$), 2.87 (s, 3H, $C–CH_3$), 3.95 (s, 3H, $N^+–CH_3$), 4.64 (t, *J* = 7.3, 2H, $N^+–CH_2–$), 7.58 – 7.66 (m, 2H, H-5, H-6), 7.93 – 8.01 (m, 1H, H-4), 8.02 – 8.10 (m, 1H, H-7).

2. NMR spectra

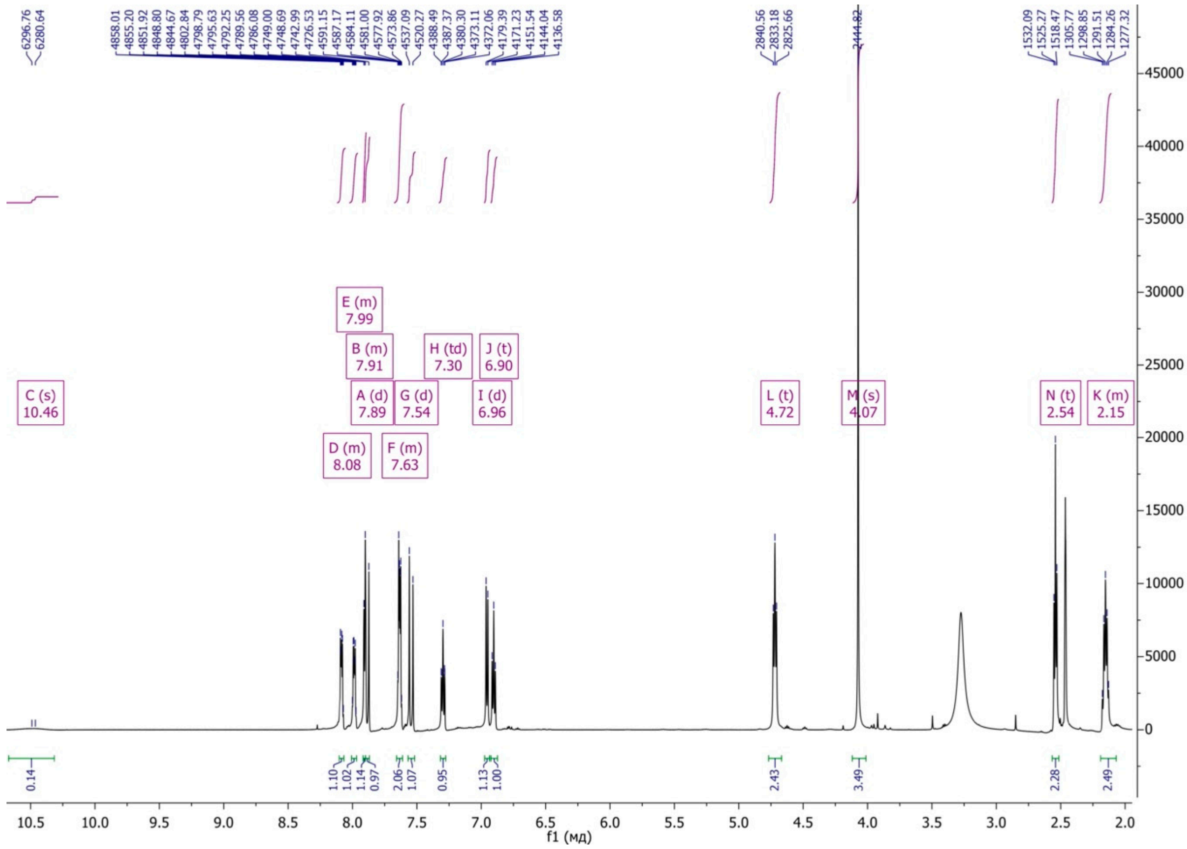

Figure S1. ^{1}H NMR spectrum of the compound **3a** in DMSO-d_6 solution.

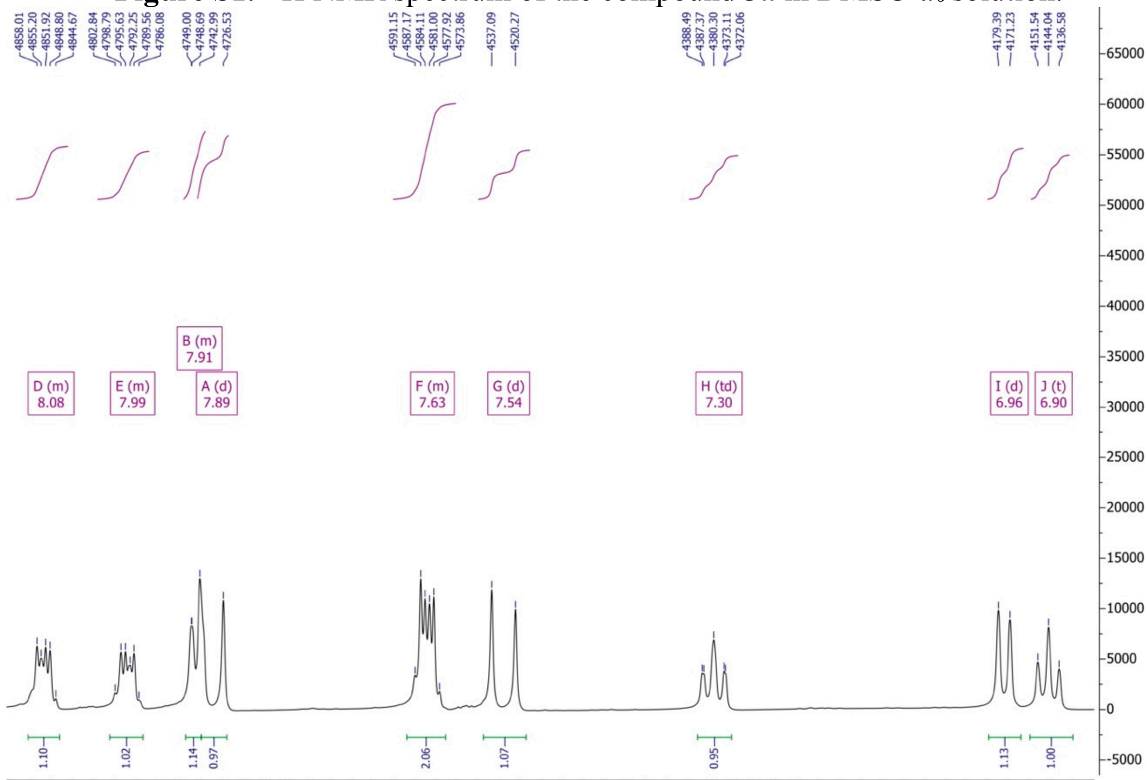

Figure S2. Aromatic region of the ^{1}H NMR spectrum of the compound **3a** in DMSO-d_6 solution.

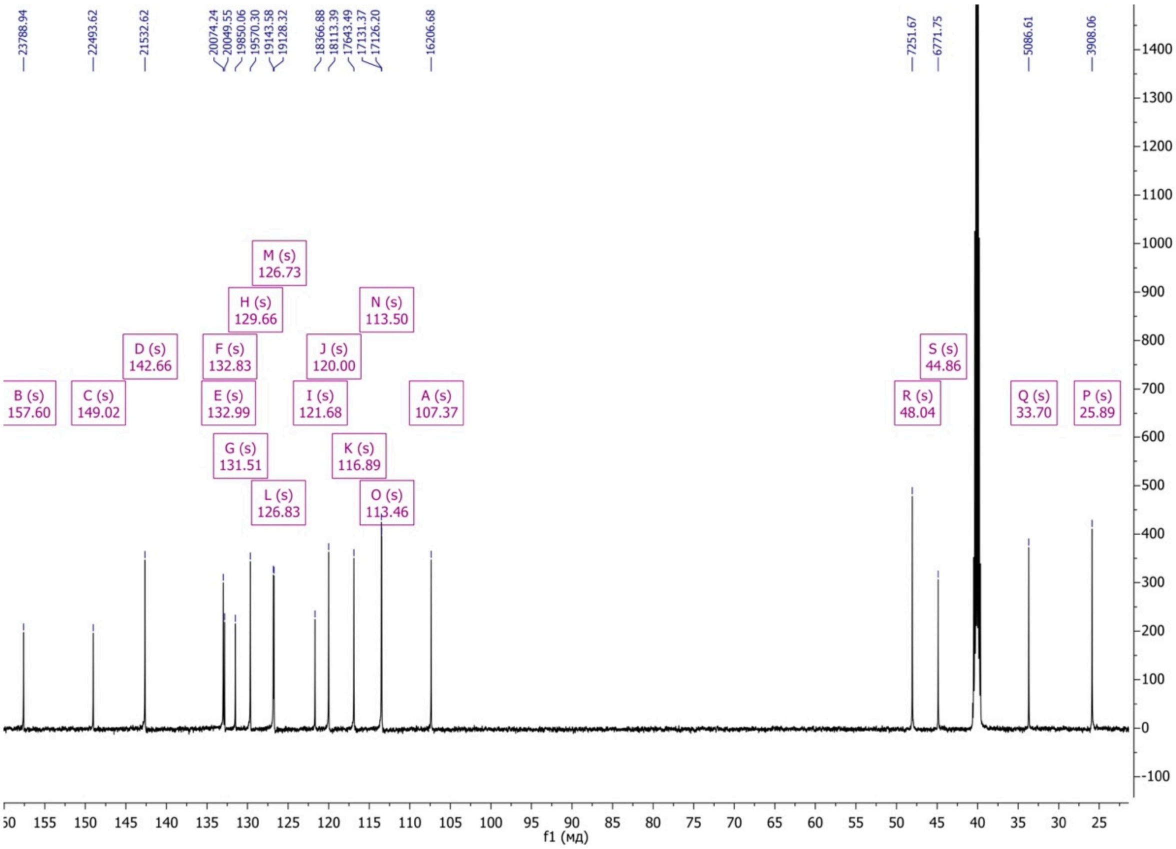

Figure S3. ^{13}C NMR spectrum of the compound **3a** in DMSO-d_6 solution.

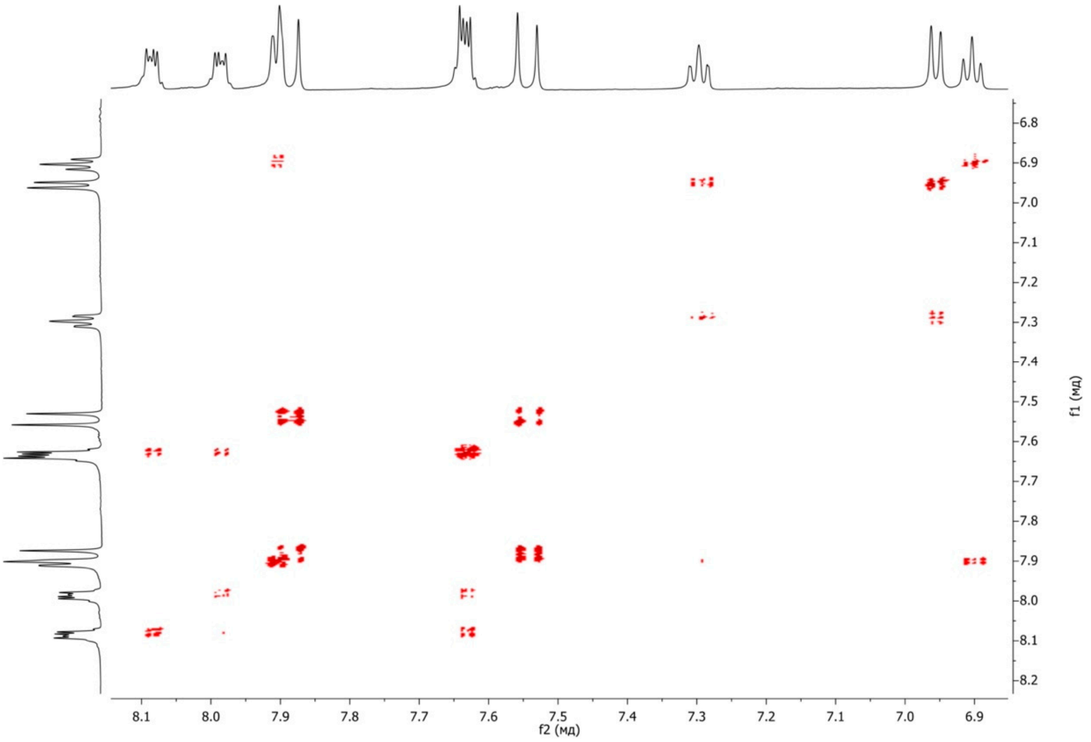

Figure S4. Aromatic region of the ^{1}H-^{1}H COSY NMR spectrum of the compound **3a** in DMSO-d_6 solution.

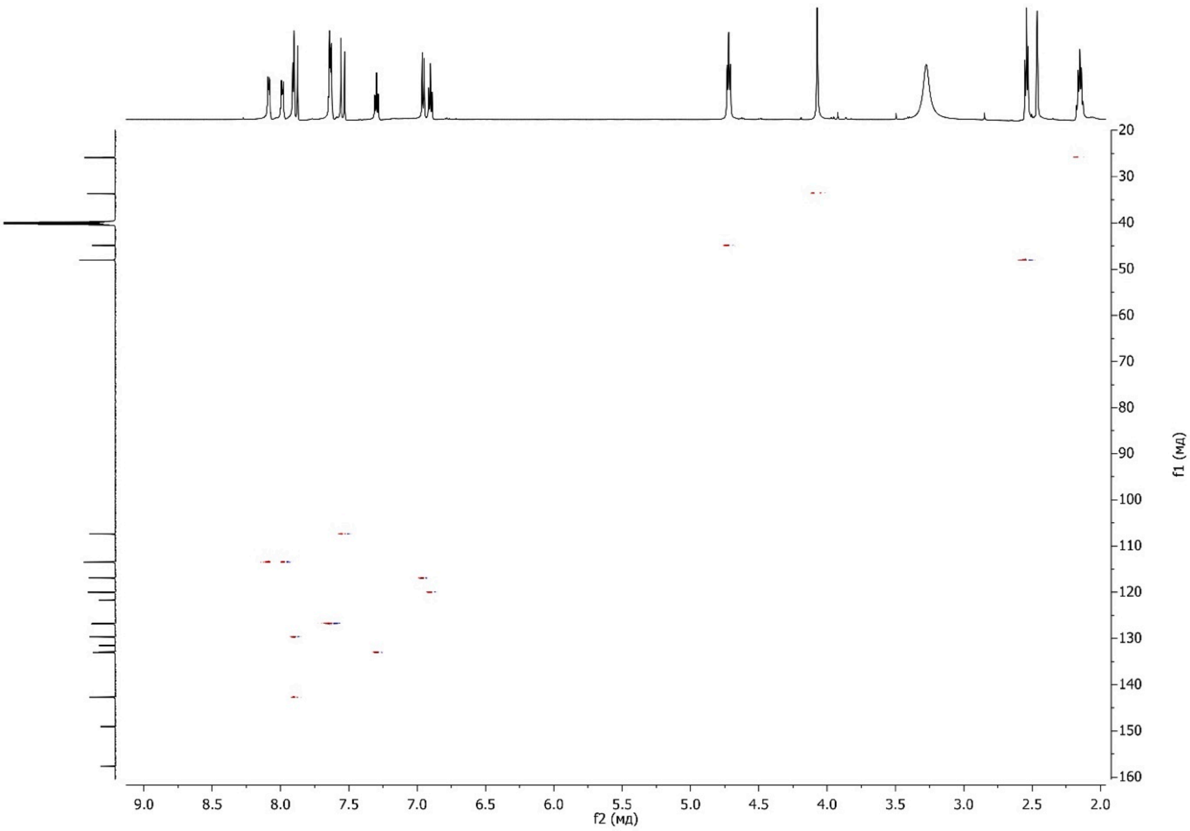

Figure S5. ^{1}H–^{13}C HSQC NMR spectrum of the compound **3a** in DMSO-d_6 solution.

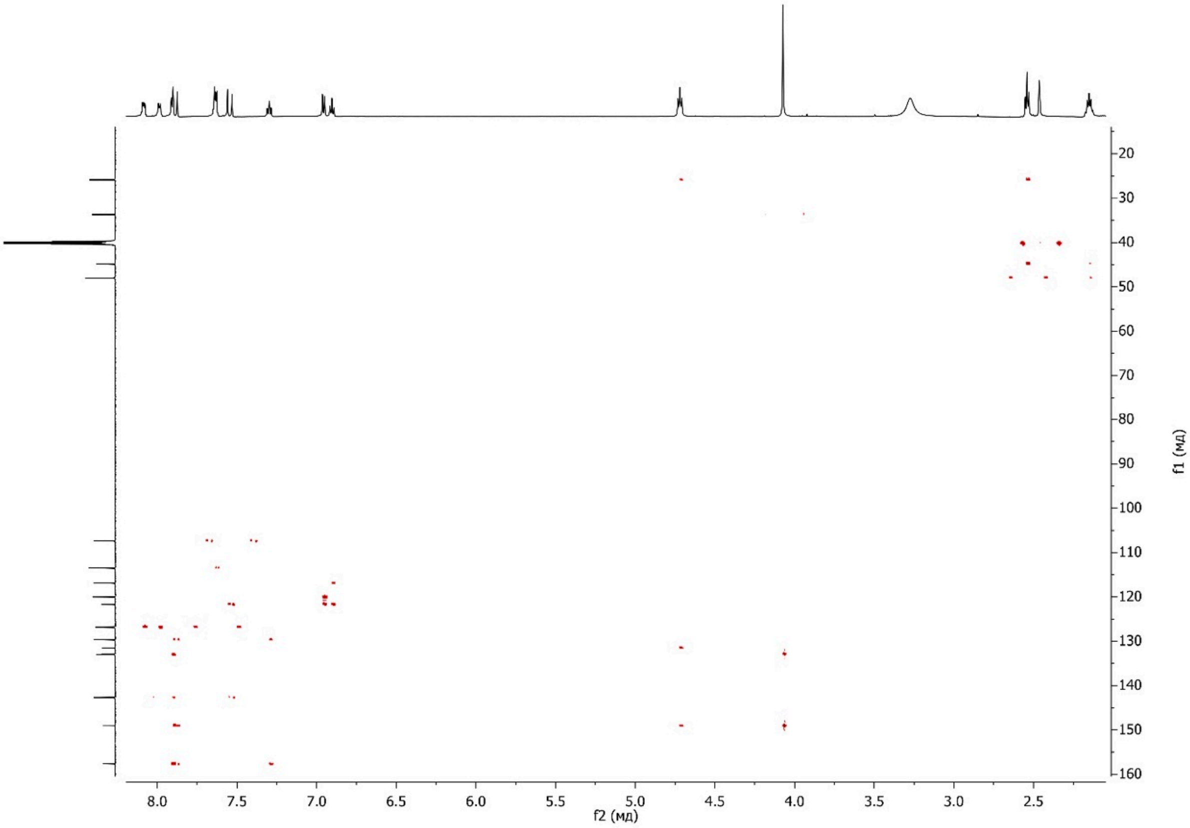

Figure S6. ^{1}H–^{13}C HMBC NMR spectrum of the compound **3a** in DMSO-d_6 solution.

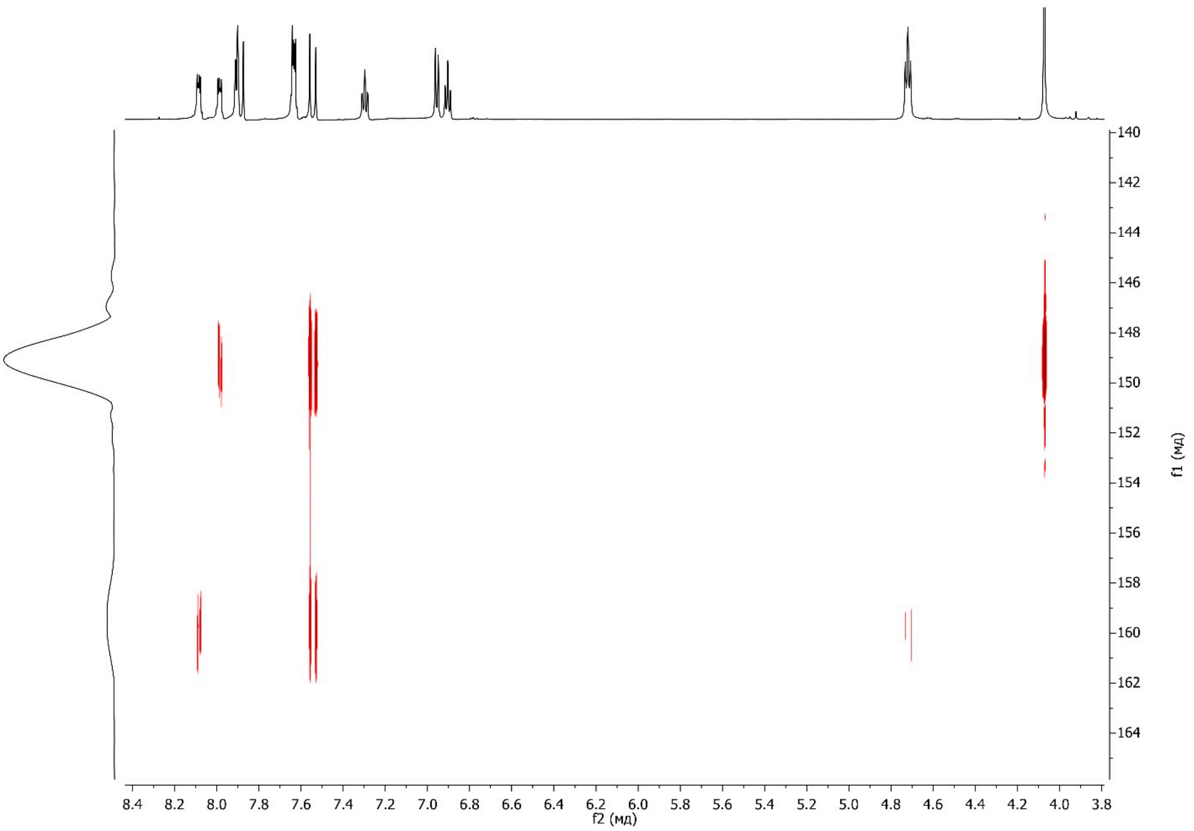

Figure S7. ^{1}H–^{15}N HMBC NMR spectrum of the compound **3a** in DMSO-d_6. solution.

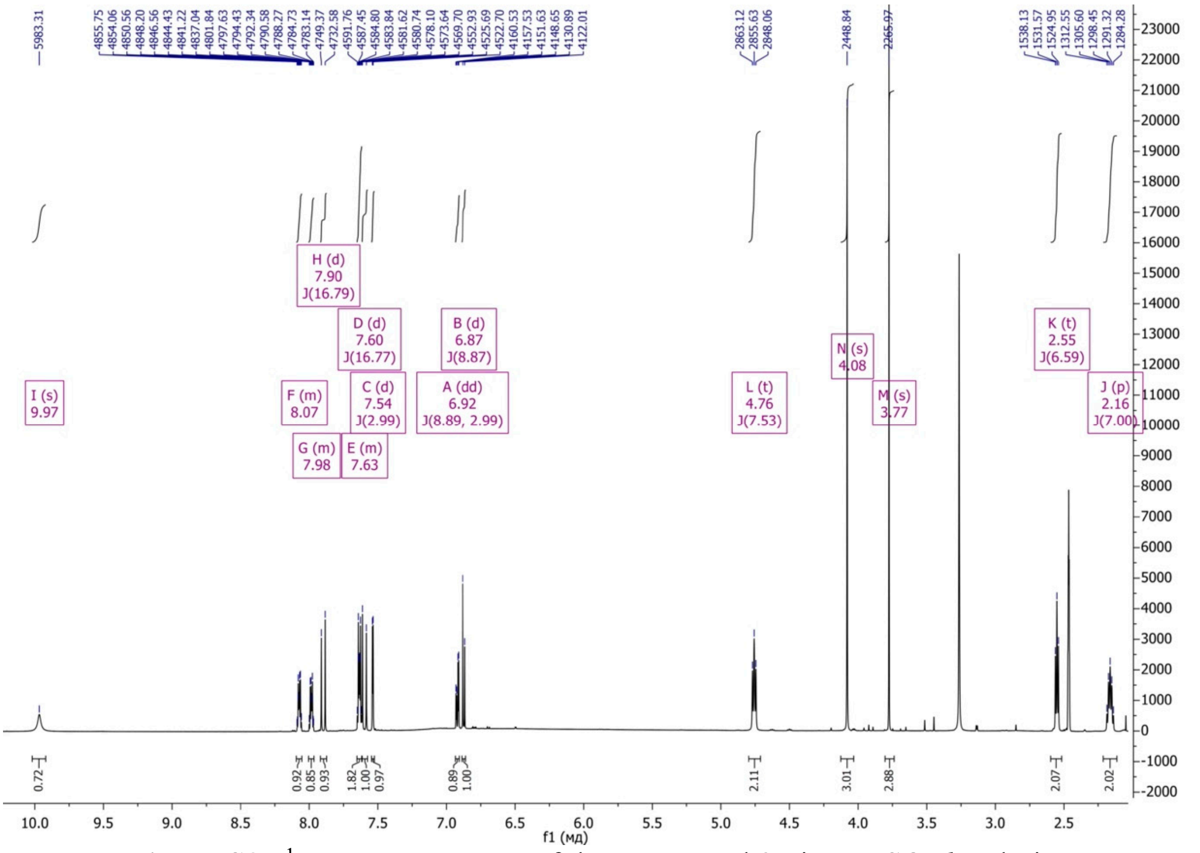

Figure S8. ^{1}H NMR spectrum of the compound **3b** in DMSO-d_6 solution.

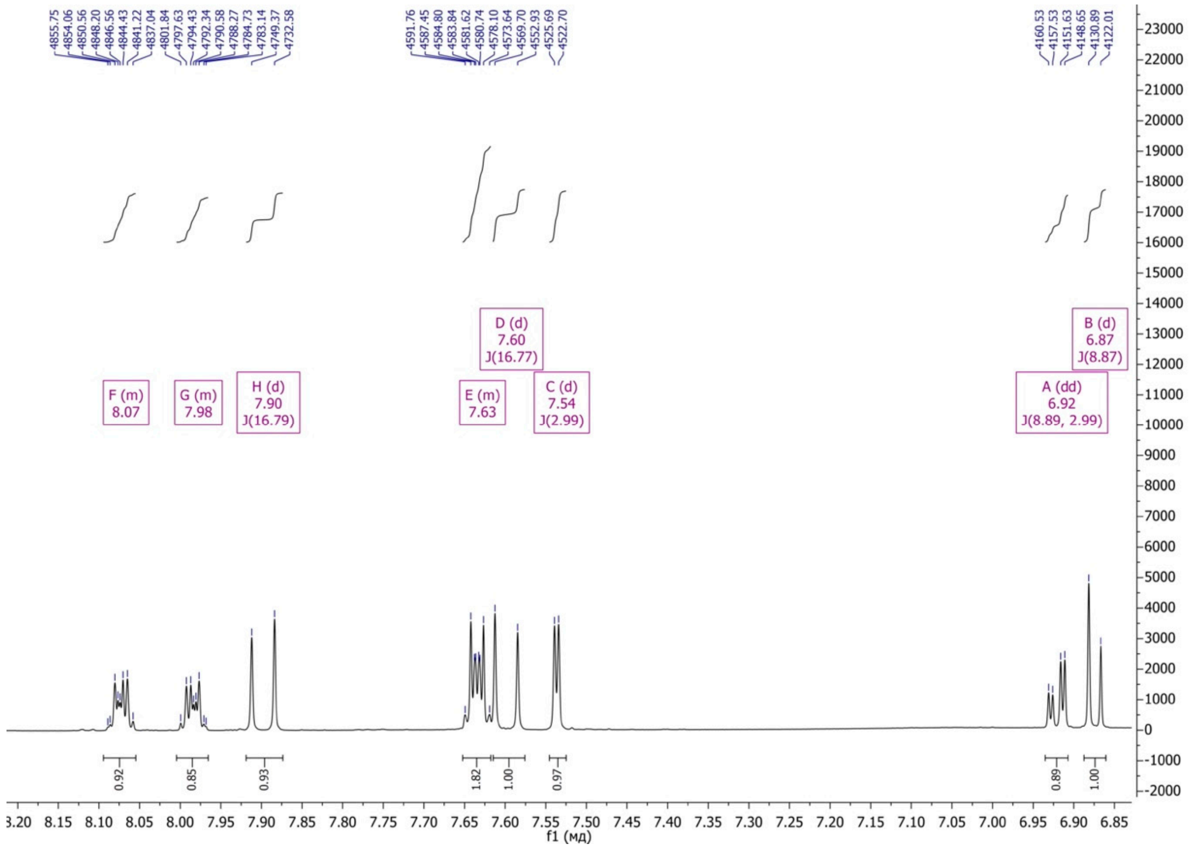

Figure S9. Aromatic region of the ^{1}H NMR spectrum of the compound **3b** in DMSO-d_6 solution.

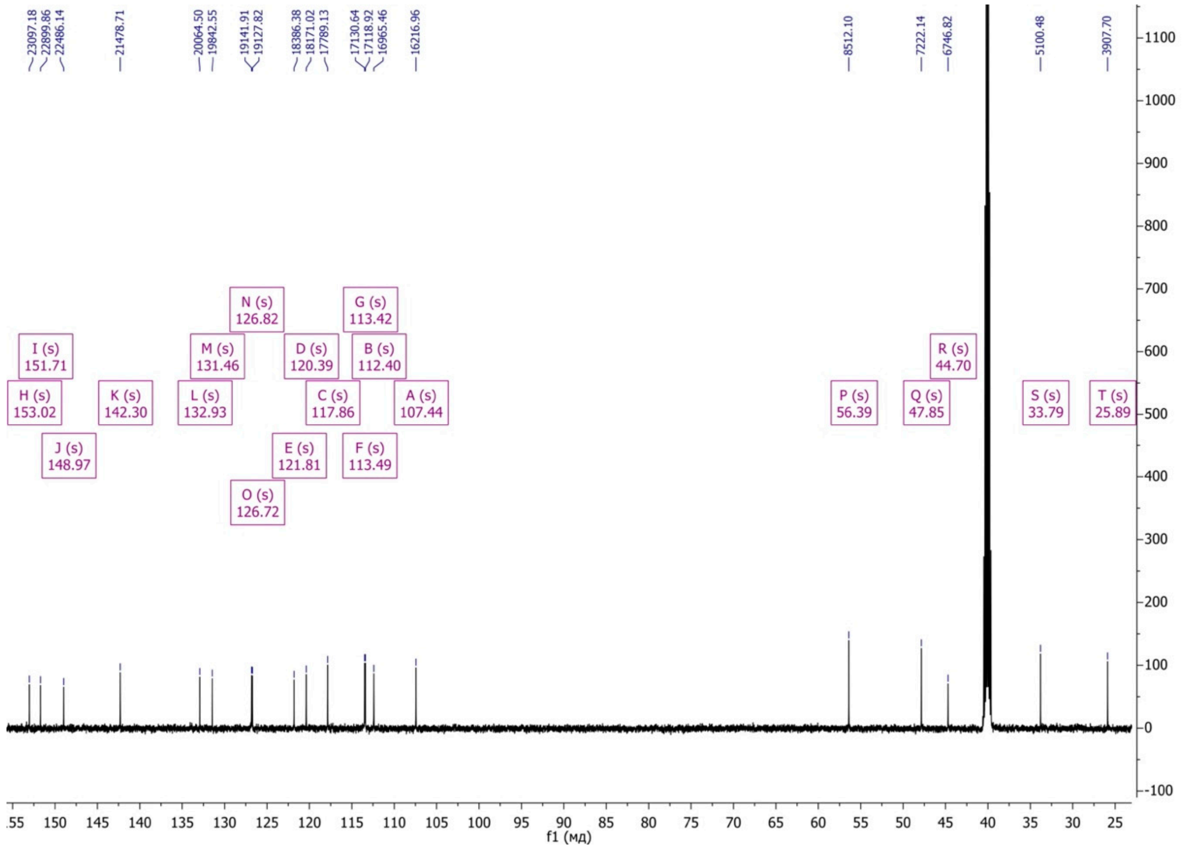

Figure S10. ^{13}C NMR spectrum of the compound **3b** in DMSO-d_6 solution.

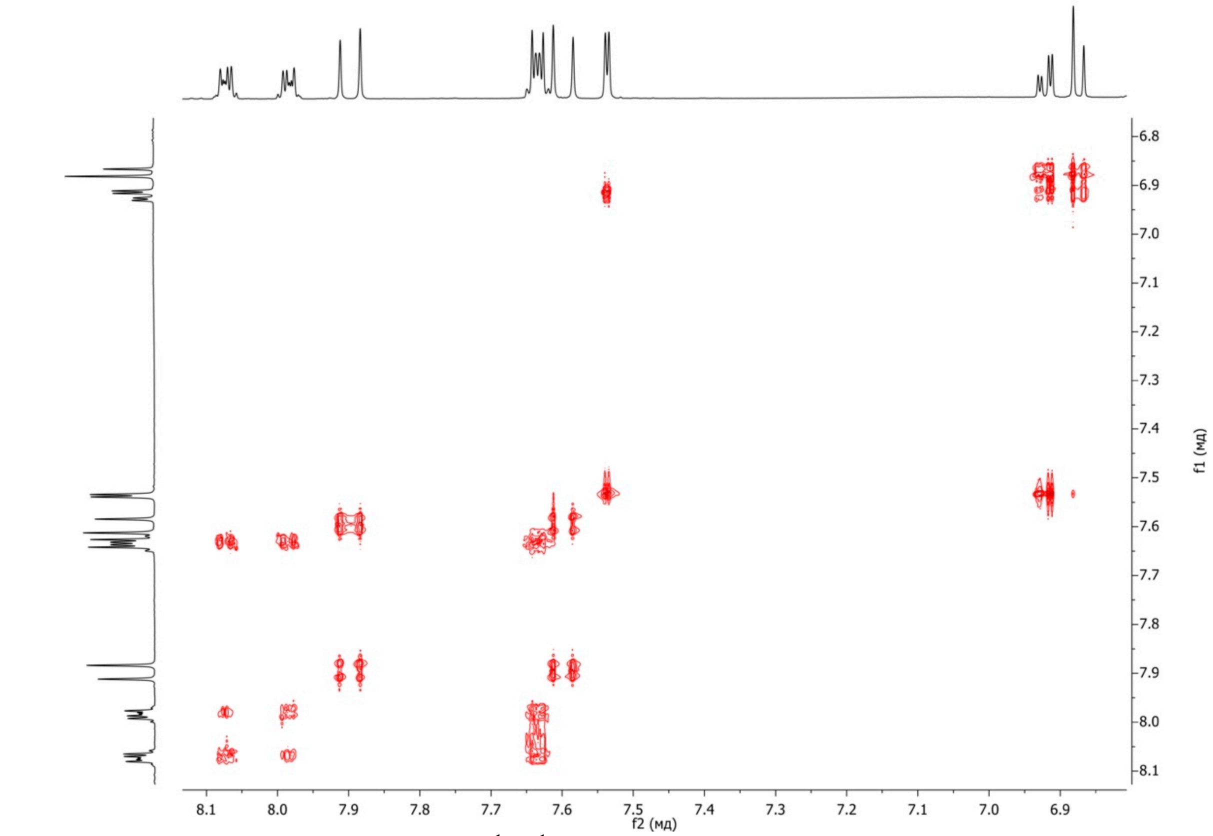

Figure S11. Aromatic region of the ^{1}H-^{1}H COSY NMR spectrum of the compound **3b** in DMSO-d_6 solution.

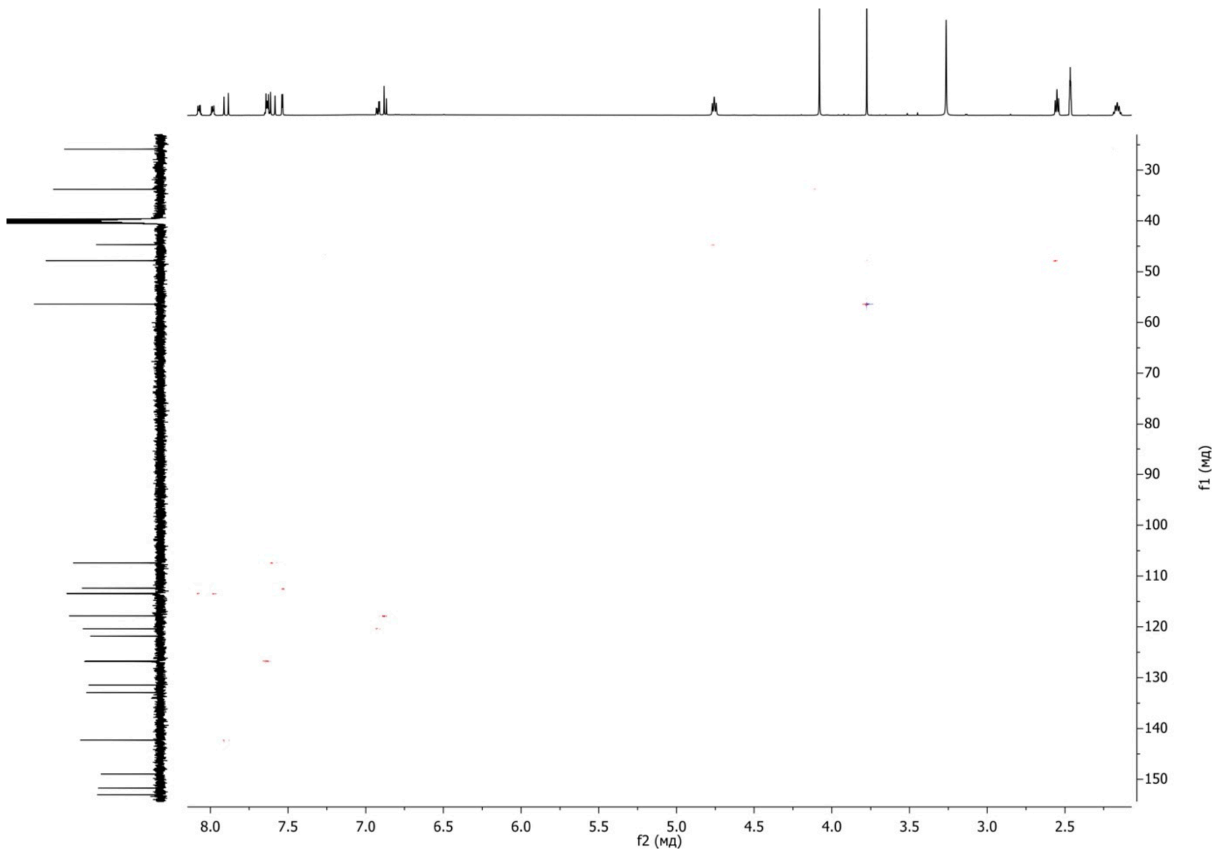

Figure S12. ^{1}H–^{13}C HSQC NMR spectrum of the compound **3b** in DMSO-d_6 Solution.

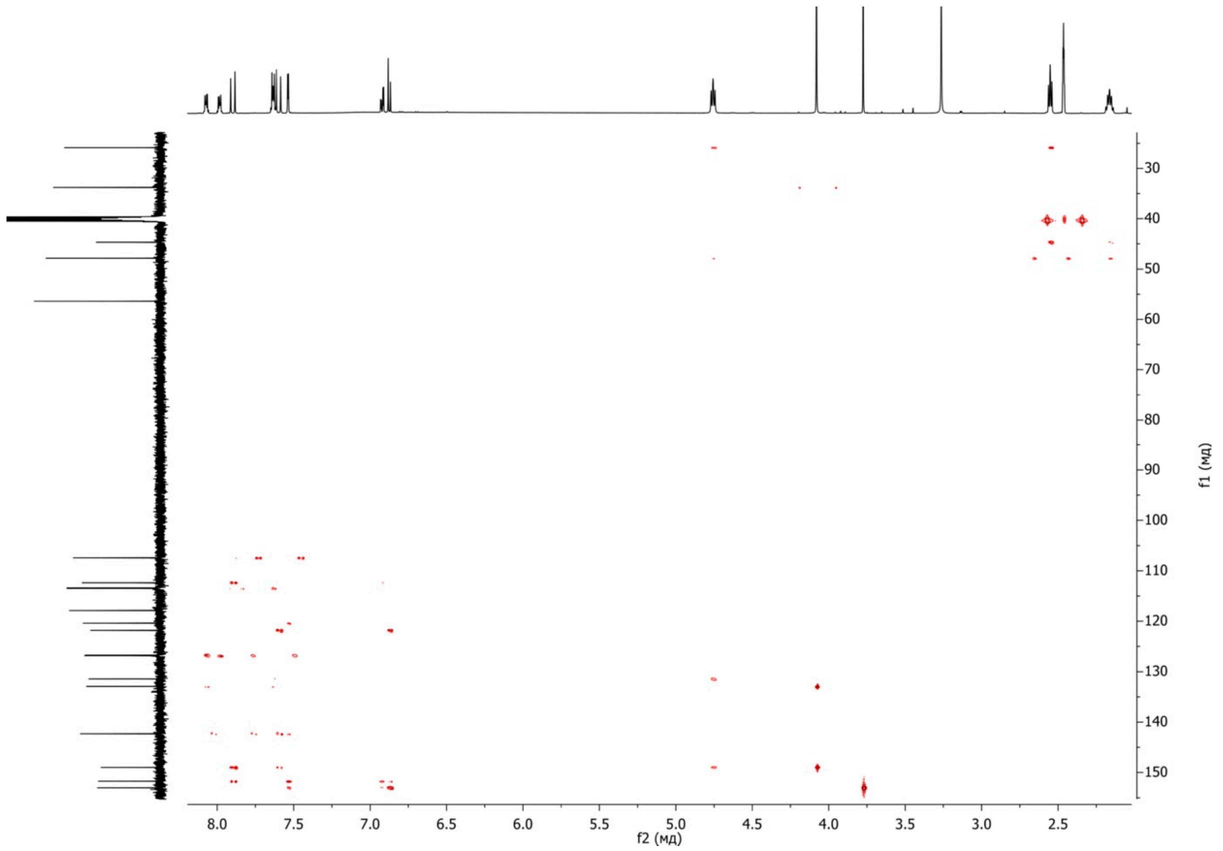

Figure S13. ^{1}H–^{13}C HMBC NMR spectrum of the compound **3b** in DMSO-d_6 solution.

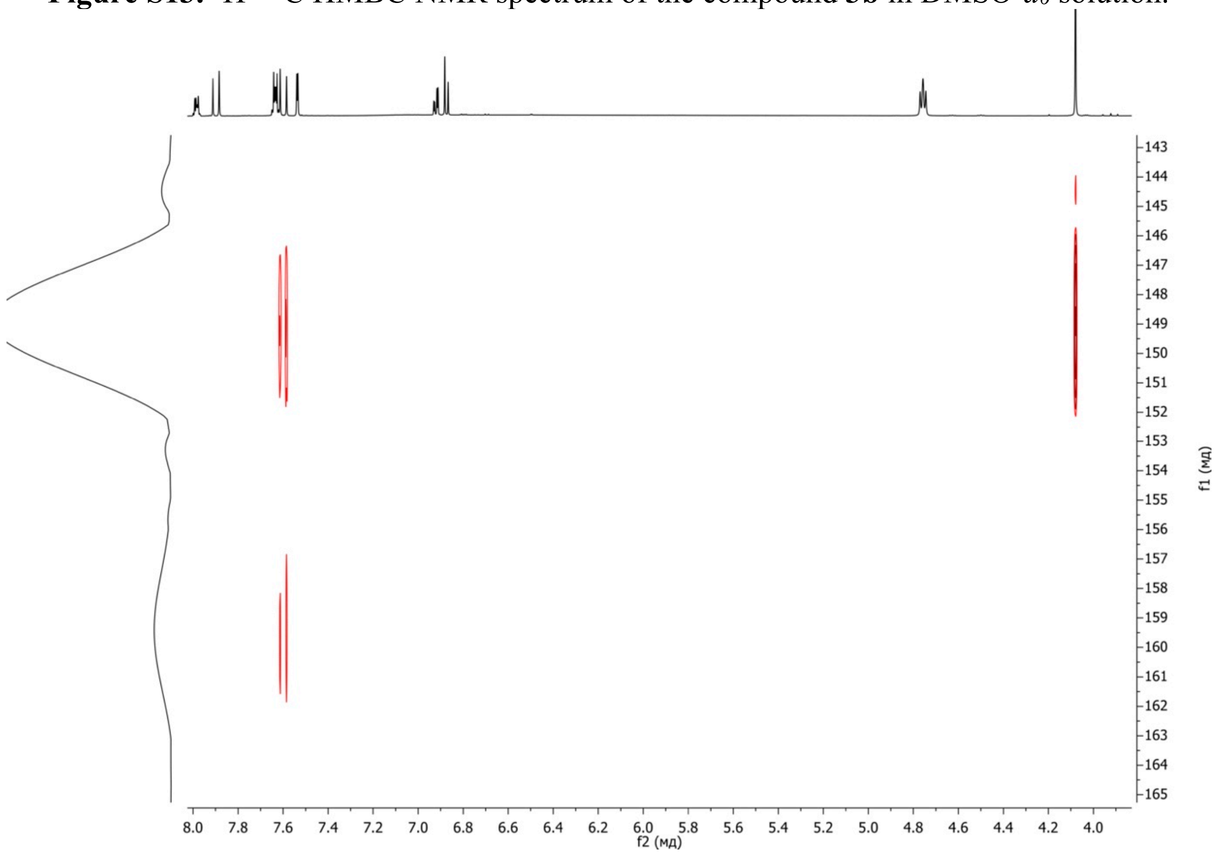

Figure S14. ^{1}H–^{15}N HMBC NMR spectrum of the compound **3b** in DMSO-d_6 solution.

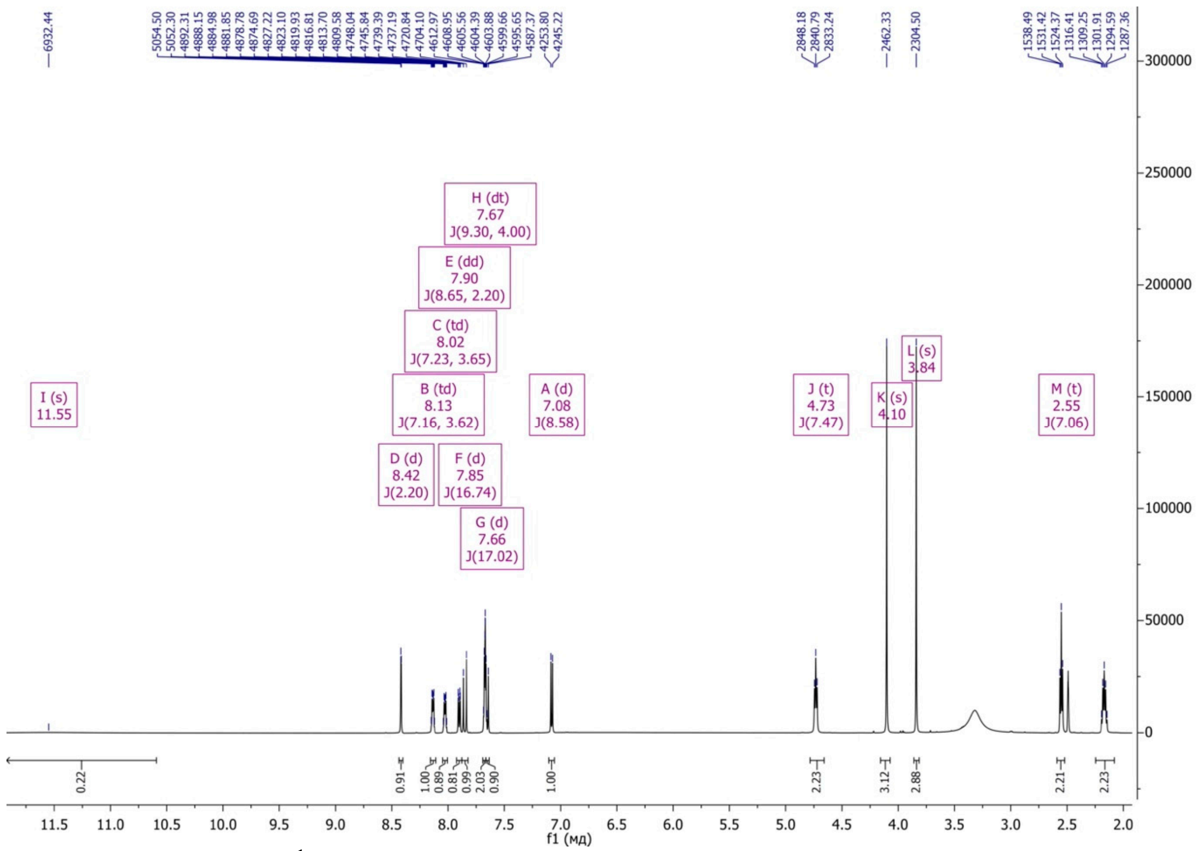

Figure S15. ¹H NMR spectrum of the compound **3c** in DMSO-*d6* solution.

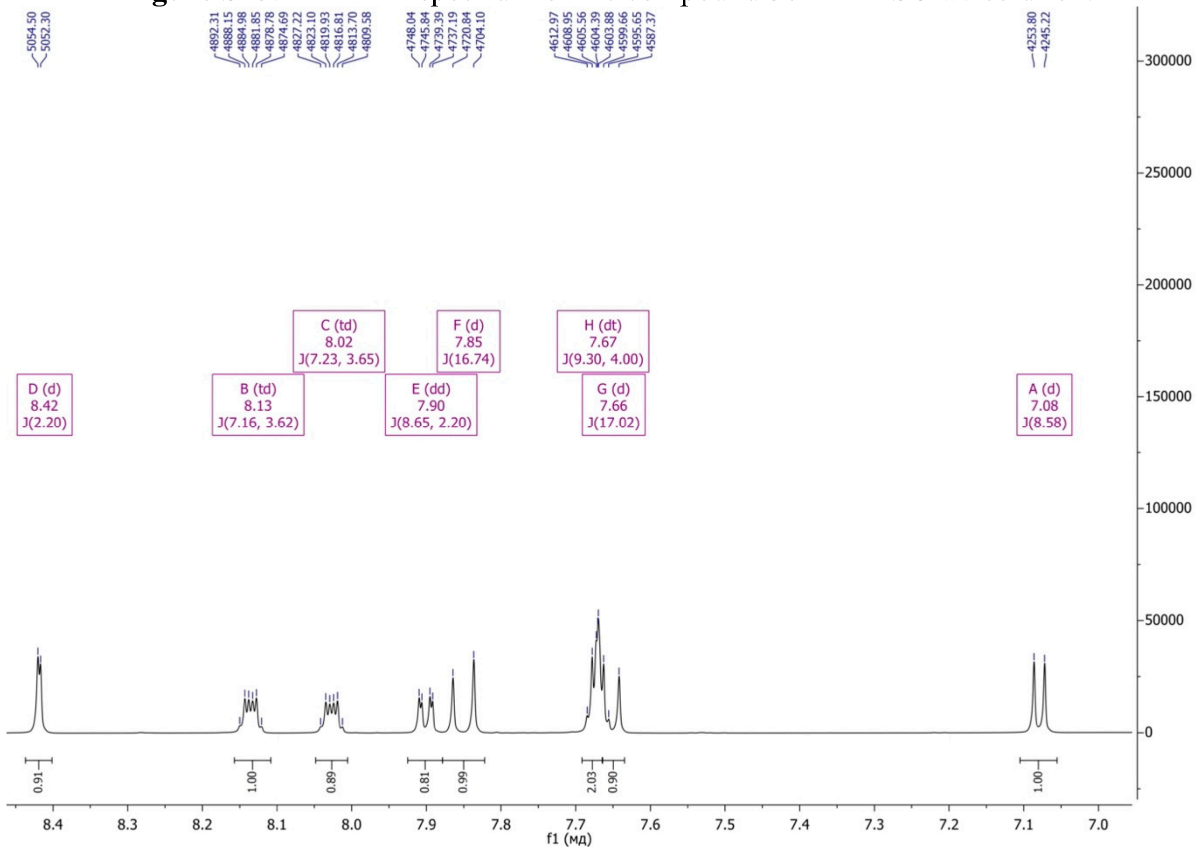

Figure S16. Aromatic region of the ¹H NMR spectrum of the compound **3c** in DMSO-d_6 solution.

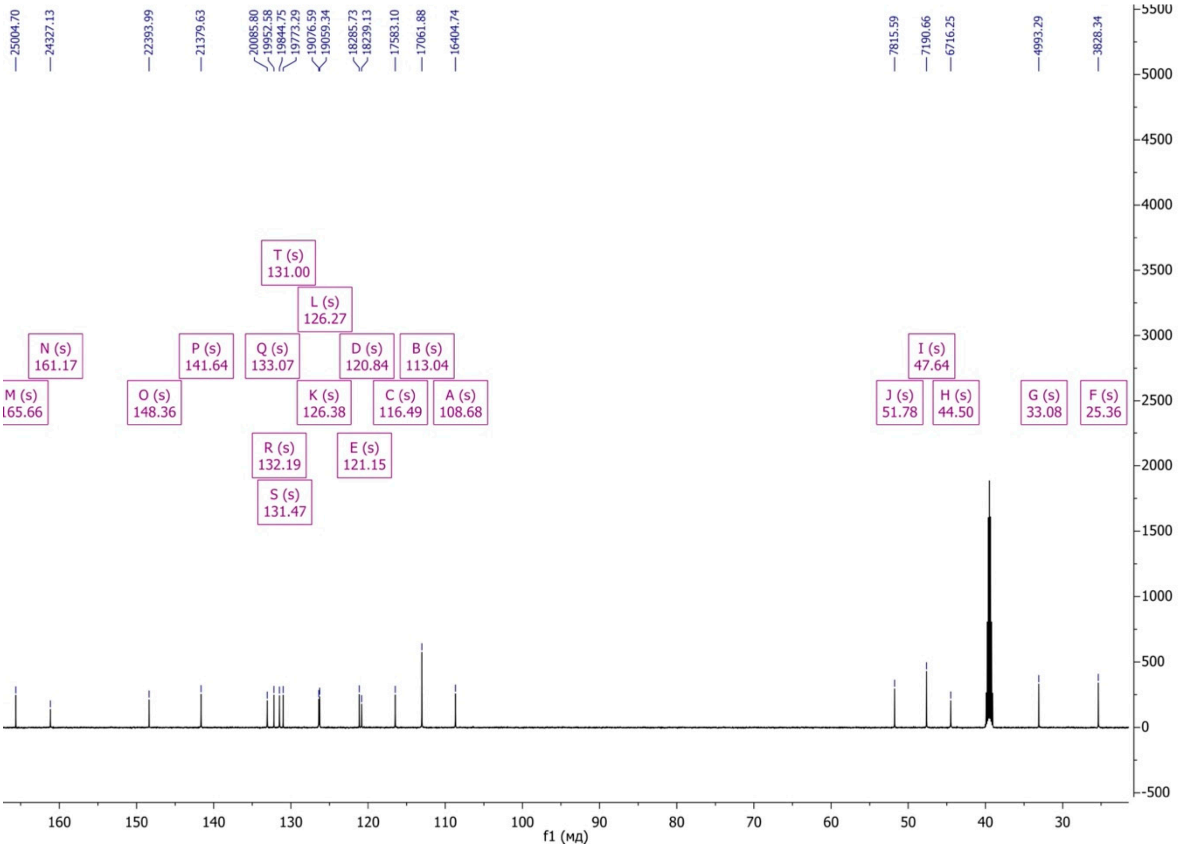

Figure S17. ^{13}C NMR spectrum of the compound **3c** in DMSO-d_6 solution.

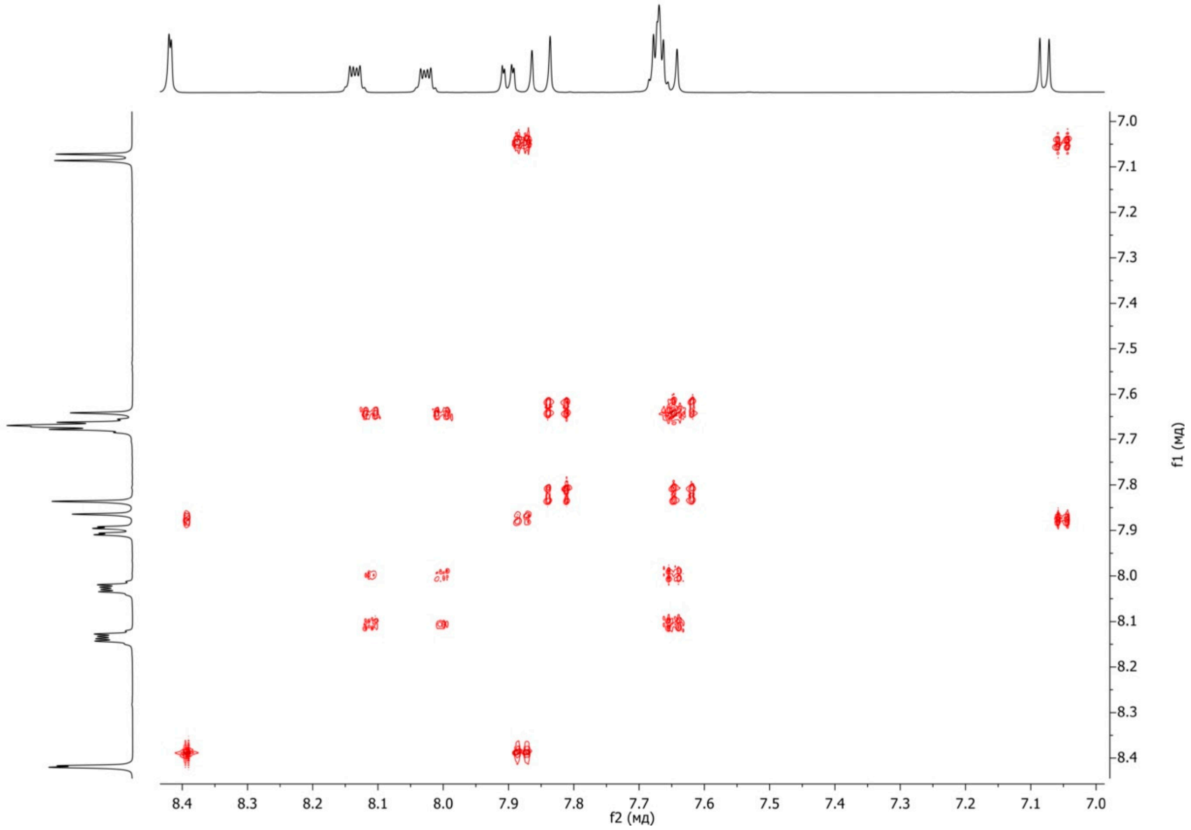

Figure S18. Aromatic region of the ^{1}H-^{1}H COSY NMR spectrum of the compound **3c** in DMSO-d_6 solution.

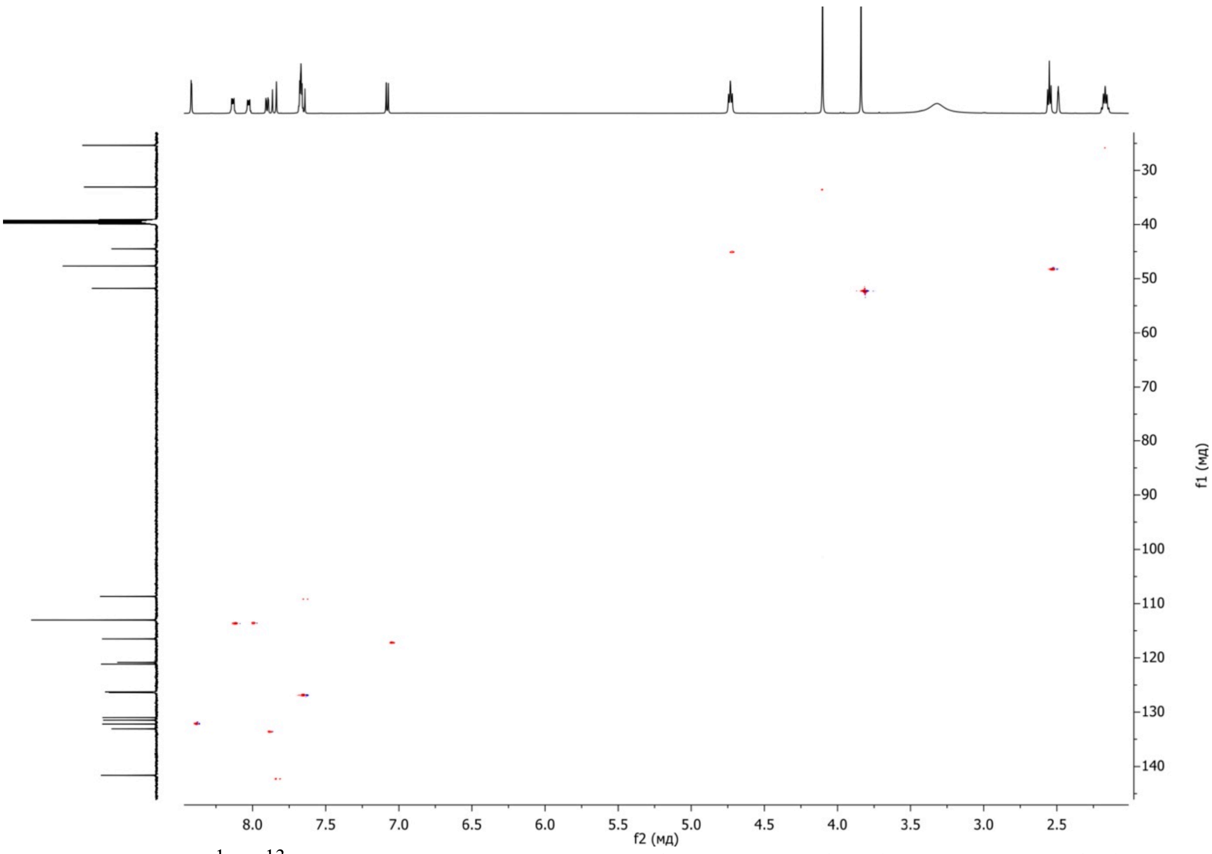

Figure S19. ^{1}H–^{13}C HSQC NMR spectrum of the compound **3c** in DMSO-d_6 solution.

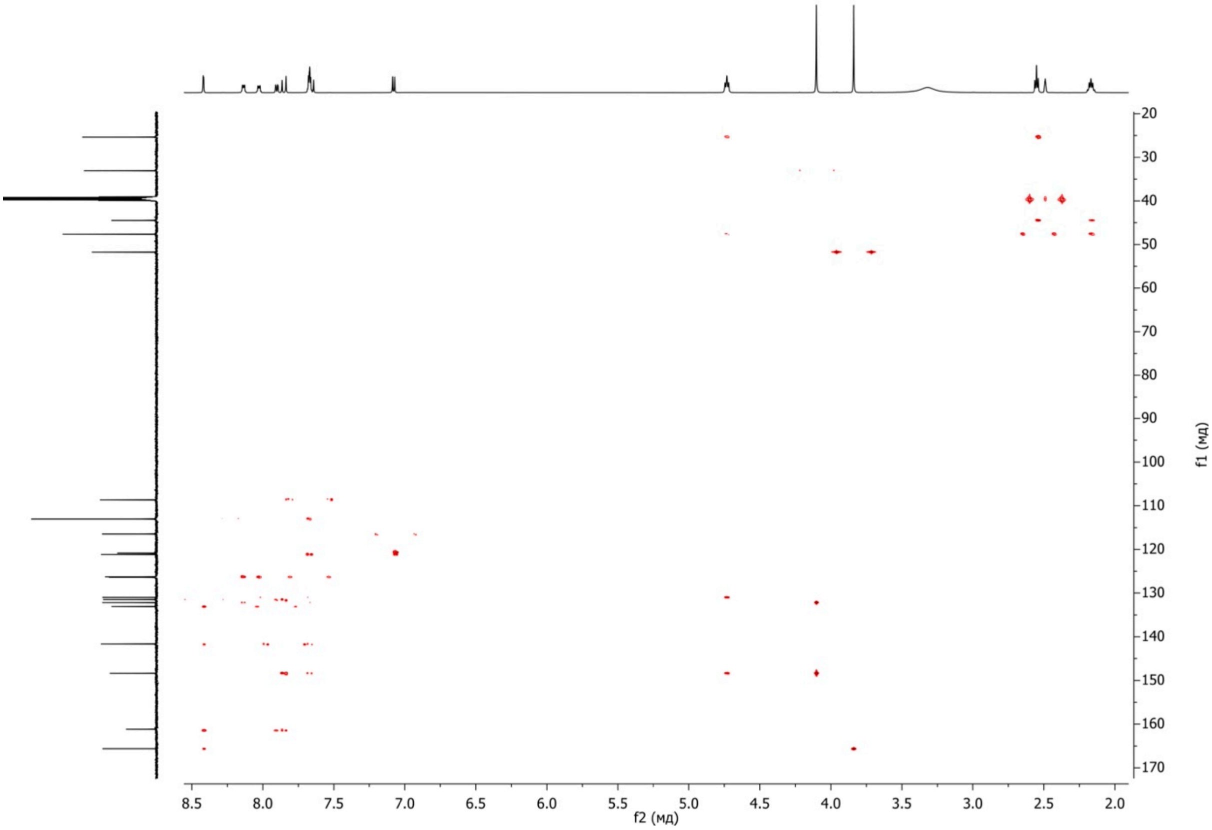

Figure S20. ^{1}H–^{13}C HMBC NMR spectrum of the compound **3c** in DMSO-d_6 solution.

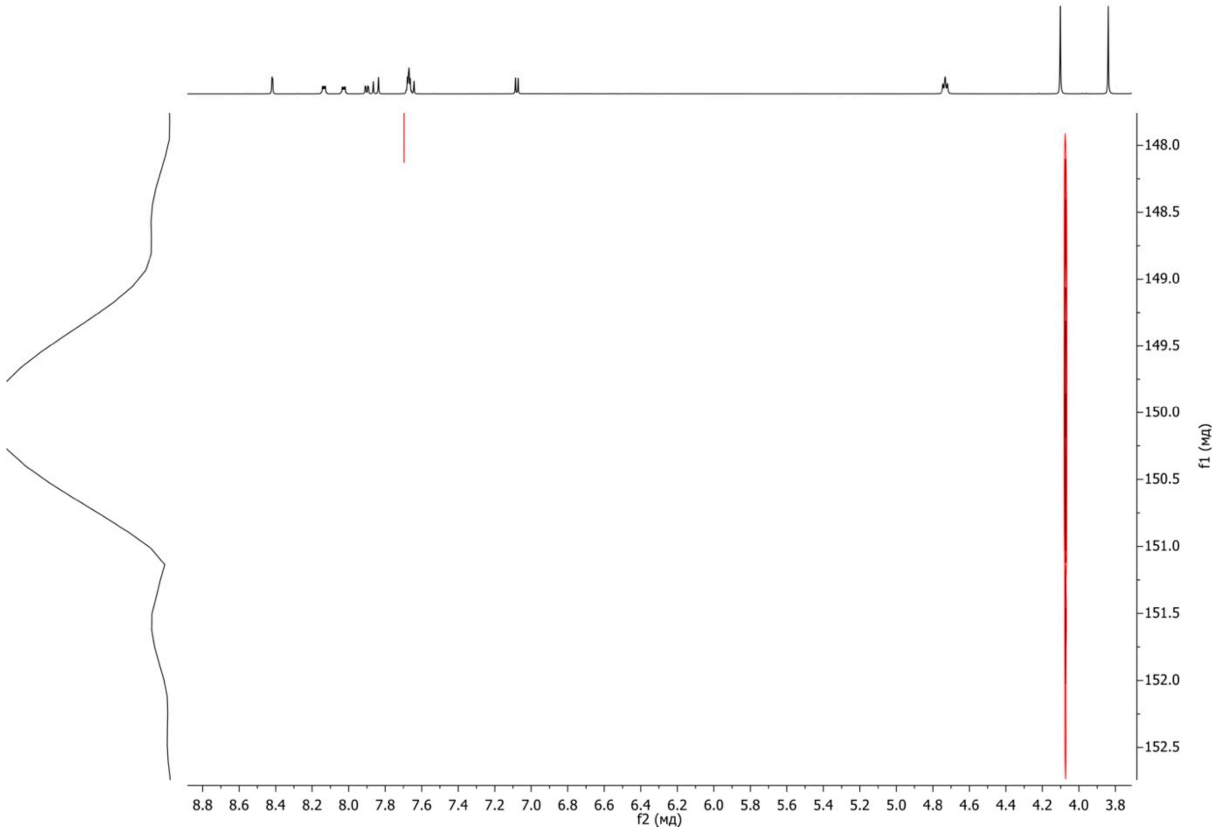

Figure S21. ^{1}H–^{15}N HMBC NMR spectrum of the compound **3c** in DMSO-d_6 solution.

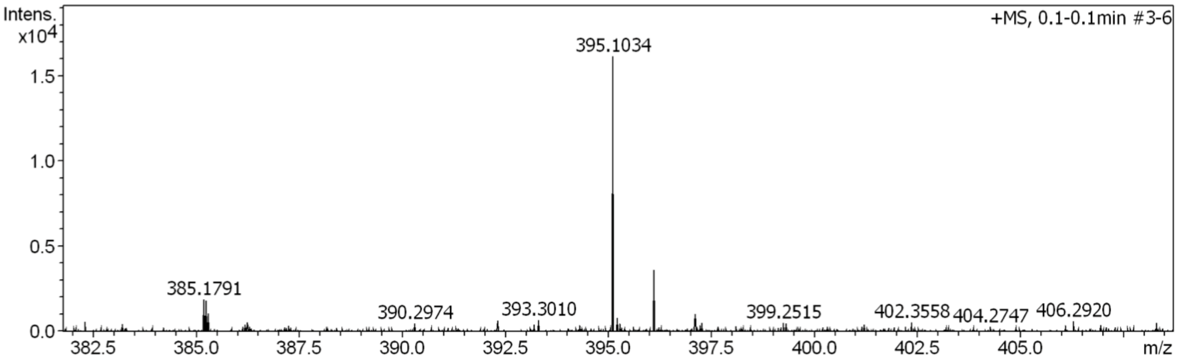

Figure S22. HRMS spectrum of **3a**.

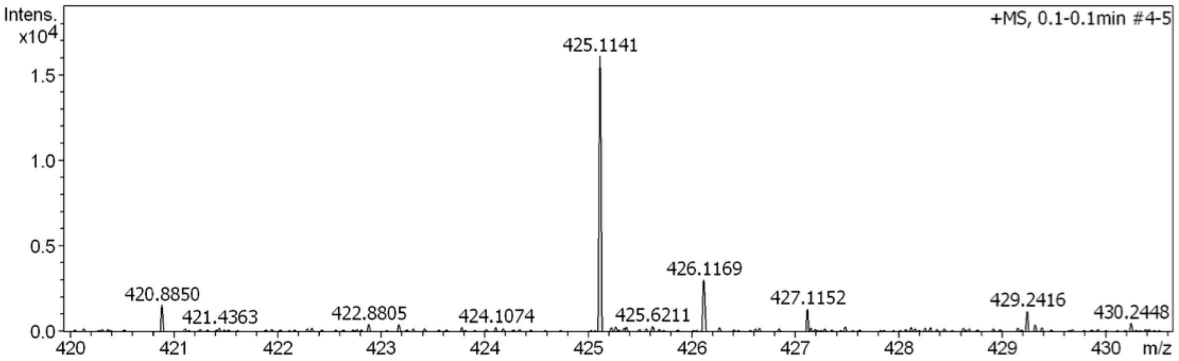

Figure S23. HRMS spectrum of **3b**.

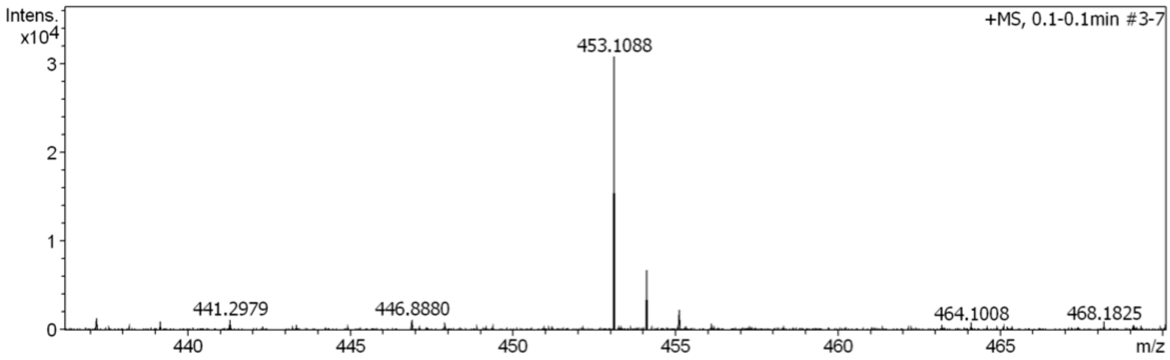

Figure S24. HRMS spectrum of **3c**.

3. Spectral studies

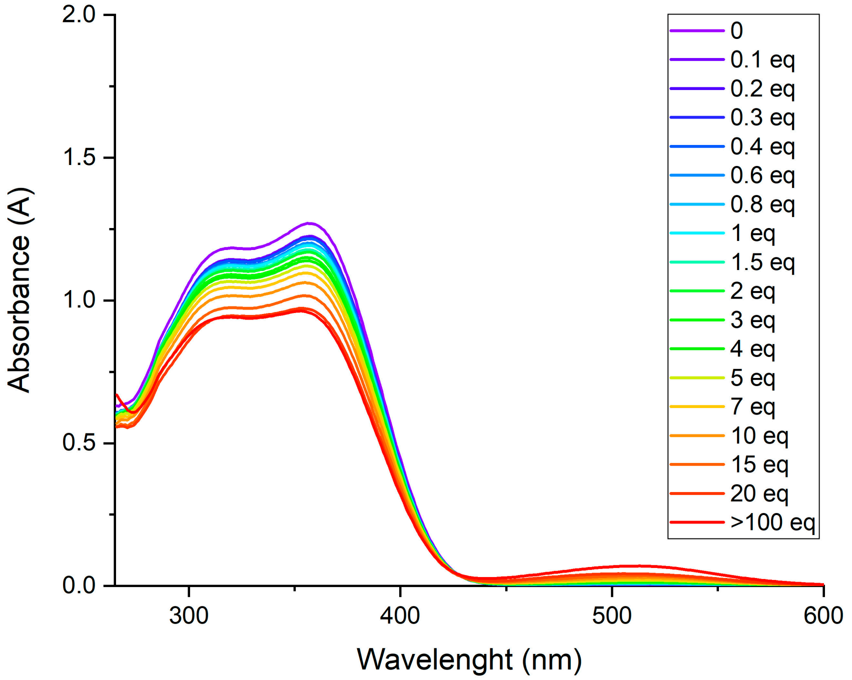

Figure S25. Absorption spectra changes of compound **3a** (100 µM) in DMSO solution during the addition of DIPEA.

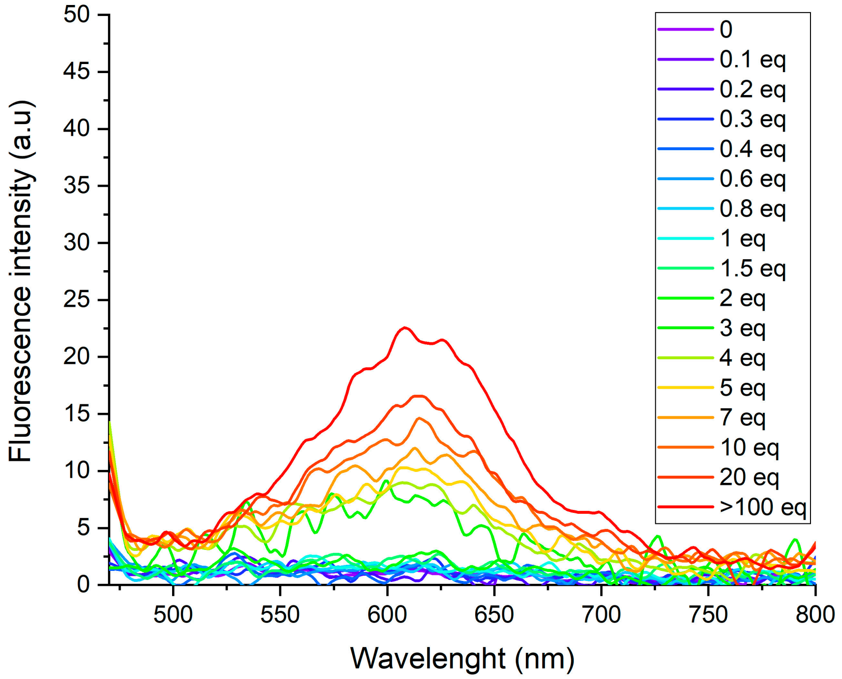

Figure S26. Photoluminescence spectra changes (λ_{ex} = 440 nm) of compound **3a** (100 µM) in DMSO solution during the addition of DIPEA.

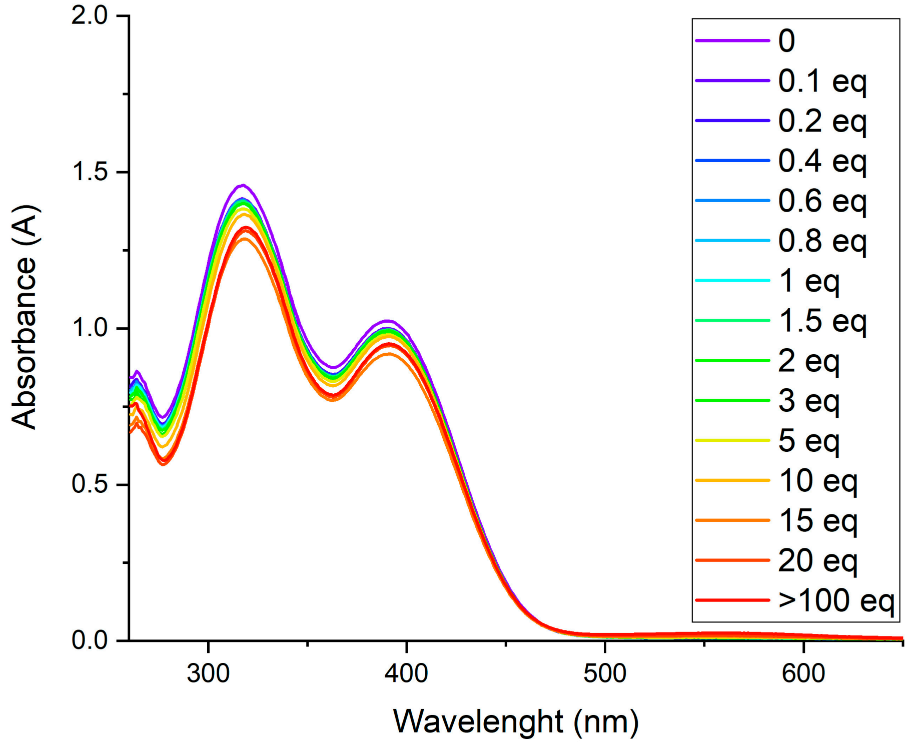

Figure S27. Absorption spectra changes of compound **3b** (100 µM) in DMSO solution during the addition of DIPEA.

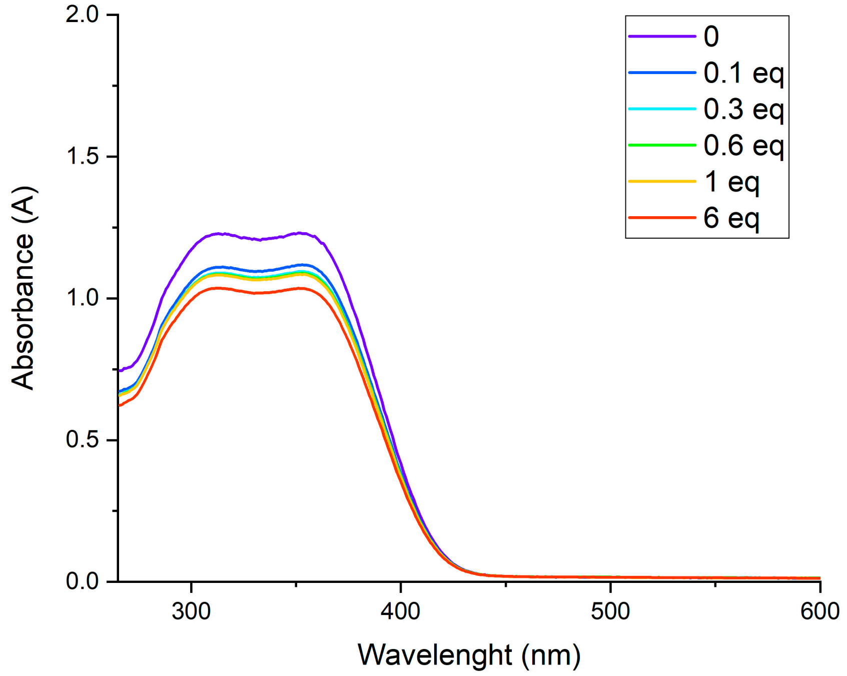

Figure S28. Absorption spectra changes of compound **3a** (100 µM) in DMSO solution during the addition of HCl.

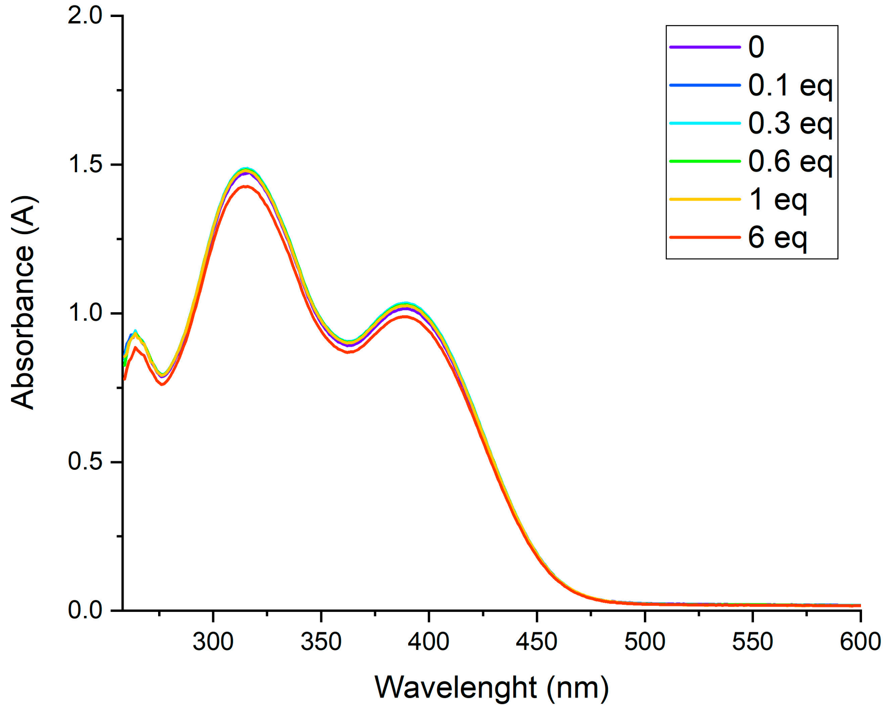

Figure S29. Absorption spectra changes of compound **3b** (100 µM) in DMSO solution during the addition of HCl.

4. Biological studies

Table S1. Reagents, used in biological assay.

Method	Reagents
Preparation of nutrient media	Peptone, bacteriological agar “Amresco”, yeast extract “Biospringer”
Assessment of toxicity by lux-biosensors	$MgSO_4 \cdot 7H_2O$, KCl, NaCl, $ZnSO_4$, KH_2PO_4(“Aquatest”), glucose-6-phosphate, NADP (“AppliChem”), S9 (“Moltox”), ethanol (“ATM Pharm”), ampicillin (“Synthesis”), benz(a)pyrene, methyl viologen (paraquat), N-methyl-N'- nitro-N-nitrosoguanidine (MNNG) (“Sigma-Aldrich). S9 was obtained from“Moltox” (USA).
Formation of biofilms	Crystal violet “Aquatest” (Russia), fluorescein diacetate (FDA) (“Sigma-Aldrich”, USA), 2,3-bis (2-methoxy-4-nitro-5-sulfophenyl)-5-[(phenylamino) carbonyl]-2H-tetrazolium hydroxide (XTT) “Aladdin” (China), dimethyl sulfoxide (DMSO) (“Serva”),ethanol (“ATM Pharm”), PBS («“VWR Life Science AMRESCO”, USA).
Preparation of nutrient media	Peptone, bacteriological agar (“Amresco”, USA), yeast extract (“Biospringer”, France).

Note: All reagents were of analytical purity.

Biofilms formation

Test system for evaluation of biofilms production. To quantify the formation of biofilms the crystal violet assay with some modifications was used [53].The necessary concentrations of the test compounds were prepared as described above.

Bacterial biofilms were grown in 96-well polystyrene plates (NUOVA APTACA, Italy). *E. coli* CDC F-50 was cultivated in LB medium [54] for 24 hours at 37 °C. The daily culture was diluted with basic mineral salt medium described before [55] with the addition of 2 % glycerol to the concentration of 1×10^8 cells/mL.

S. aureus ATCC 6538-P FDA 209-P was cultivated in LB medium for 24 hours at 37 °C. The daily culture was diluted with LB medium to the concentration of 1×10^8 cells/mL.

180 µl of microorganism suspension was placed into the wells of the plates. To some of the wells, 20 µl of the test substances at various concentrations were added. A sterile basic mineral salt medium served as a negative control. Part of the wells was used as a positive control; since solvents used could also influence the biofilm formation, 20 µl of the appropriate solvent was added to the other part of the wells at same dilutions. The plates were

covered, wrapped in parafilm (Bemis Company, United States) and placed into a thermostat for 24 hours at 37 °C for *E. coli* CDC F-50 and at 37 °C for *S. aureus* ATCC 6538-P FDA 209-P. Staining was performed after incubation.

Biofilms were stained with crystal violet (CV) to determine the biomass [53, 56]. The optical density of the dye bound to the biofilm was determined spectrophotometrically at a wavelength of 570 nm using a FLUOstar Omega plate reader (BMG LABTECH, Germany). The intensity of biofilm formation (%) was calculated by the formula: $(T\text{-}B)/(C\text{-}B)\cdot 100$ %, where T is the optical density of the experiment, B is the optical density of the negative control, C is the optical density of the positive control.

Analysis of viable cells number. Fluorescein diacetate staining was used to analyze the number of viable cells [57, 58]. Fluorescence intensity measurement was performed using a FLUOstar Omega plate reader (λ_{ex}: 485±12 nm and λ_{em}: 520±10 nm). The number of living cells was evaluated in relation to the positive control, which was taken as 100%.

Metabolic activity of biofilm cells. The XTT reductase test method was used to determine the metabolic activity of cells [58 – 60]. Optical density was measured at 486 nm using a FLUOstar Omega plate reader. The level of metabolic activity was calculated in relation to the positive control, which was taken as 100%.

Each experiment was performed in triplicate and repeated in six different occasions. The values were expressed as mean ± SD. Student’s T-test was used to compare these values. Differences were considered statistically significant at $p < 0.05$.

Assessment of substances toxicity using lux-biosensors

Inducible whole-cell luminescent bacterial biosensors *E. coli* MG1655 (pRecA-lux), *E. coli* MG1655 (pAlkA-lux) were used to detect genotoxic effects [61, 62]. To determine promutagens, S9 rat liver microsomal enzyme fraction was used (Moltox, the USA).

E. coli MG1655 (pKatG-lux) and *E. coli* MG1655 (pSoxS-lux) biosensors were used that specifically respond to oxidative stress-inducing substances in the cell. *E. coli* MG1655 (pKatG-lux) reacts to the production of hydroperoxides in a cell. *E. coli* MG1655 (pSoxS-lux) responds to superoxide anion and NO [62].

The *E. coli* MG1655 (pIbpA-lux) biosensor was used to detect substances that cause protein damage in cells. The study of samples for integral toxicity was carried out using the strain *Vibrio aquamarinus* VKPM B-11245 (*V. aquamarinus* DSM 26054) [63, 64].

To correct artifacts associated with changes in bacterial luciferase activity and not associated with induction, a strain with a constitutive promoter *E. coli* MG1655 (pXen7) was used [62]. *The Photorhabdus luminescens* gene cassette luxCDABE under the control of Plac,

PrecA, PcolD, Palk, promoters, respectively, was used in these biosensors. The plasmids were constructed based on the pBR322 vector and contain a selective ampicillin resistance marker.

Bacterial strains were cultivated in Luria-Bertani medium, supplemented with 100 µg of ampicillin mL^{-1}, at 37 °C overnight. Then the cultures were inoculated to optical densities (OD590) of 0.025 from an overnight culture and cultivated at 37 °C for 2.5 h to OD590 of approximately 0.2 determined by FLUOstar Omega microplate reader (BMG Labtech, Germany). Cells were used immediately for stress induction tests. The tested substances were added in 10 µL portions to wells of a 96-well microplate containing 190 µL of the culture. In the control, 10 µL of extractant was added. 10 µL of toxicant solution (in case of positive control for promoter activation) was introduced into other wells. Luminescence was measured with Luminoscan Ascent microplate luminometer (Thermo Fisher Scientific, the USA) in three independent replications. Toxic influence of the studied substances on genetically engineered *E. coli* strains was measured according to the induction of their bioluminescence for 120-min exposition period. The degree of luminescence induction evaluated using the induction factor (*I*) calculated as follows:

$$I = \frac{L_c \times l_k}{L_k \times l_c} \quad (1)$$

where L_c is the luminescence intensity of a lux-biosensor suspension, containing tested sample, L_k is the luminescence intensity of a lux-biosensor control suspension, l_c is the luminescence intensity suspension lux-strain with constitutive promoter (*E. coli* MG1655 (pXen7)) in the presence of the test compound, and l_k is the luminescence intensity control suspension lux-strain with constitutive promoter (*E. coli* MG1655 (pXen7)).

The detected toxic effect for the genetically engineered *E. coli* strains according to the *I* values was evaluated as follows: weak toxicity ($I < 2$), moderate toxicity ($2 \le I \le 10$), and strong toxicity ($I > 10$).

Strong toxic influence of the studied toxicant on *V. aquamarinus* was evaluated according to the inhibition of its bioluminescence for 30-min exposition period. Toxicity index (*T*) was used to determine integral toxicity of the samples defined as:

$$T = \frac{100 \times (I_k - I_c)}{I_c} \quad (2)$$

where I_c and I_k are the intensity of bacteria luminescence in tested and control samples, respectively, at fixed exposition time of the studied solution with test object. The

following toxicity classes were estimated using *V. aquamarinus:* admissible (I < 20), toxic (20 ≤ I < 50), and highly toxic (I > 50).

All the experiments were carried out three times independently. The differences from control were considered statistically significant at $p < 0.05$. Statistical analysis was performed using GraphPad Prism 8.0.2 DEMO (GraphPad Software, Inc., SanDiego, CA) using two-way ANOVA and t-test ($p \leq 0.05$).

Table S2. Effect of compounds **3a-c** on biomass, the level of metabolic activity, and the number of living cells in the bacterial biofilm of the *E. coli* CDC F-50

Concentration of **3a-c**	Intensity of biofilm formation, OD 570 nm		Metabolic activity of the biofilm, OD 486 nm		Number of viable cells, Fluorescence intensity λ_{ex} = 485 ± 12 nm, λ_{em} = 520 ± 10 nm	
	**3a**	control	**3b**	control	**3c**	control
10^{-8}	0.097±0.009	0.089±0.008	0.010±0.006	0.023±0.007	1064±122*	1522±220
10^{-7}	0.081±0.009	0.086±0.011	0.015±0.003*	0.040±0.009	979±78*	1557±127
10^{-6}	0.100±0.006	0.097±0.004	0.045±0.014	0.047±0.017	966±25*	1461±200
10^{-5}	0.095±0.008	0.090±0.004	0.013±0.003*	0.055±0.011	1130±54*	1639±301
10^{-4}	0.079±0.007*	0.116±0.001	0.040±0.009*	0.117±0.018	1371±89*	1975±392
	**3a**	control	**3b**	control	**3c**	control
10^{-8}	0.075±0.008	0.084±0.008	0.040±0.009*	0.018±0.004	5534±523	5491±317
10^{-7}	0.073±0.004	0.080±0.007	0.074±0.004*	0.033±0.008	5802±334*	5171±161
10^{-6}	0.072±0.003*	0.090±0.010	0.059±0.008*	0.040±0.009	6065±361	5972±241
10^{-5}	0.080±0.002	0.085±0.010	0.078±0.009*	0.060±0.008	5647±535	5477±448
10^{-4}	0.081±0.006*	0.143±0.026	0.051±0.011*	0.110±0.012	6160±415	6105±429
	**3a**	control	**3b**	control	**3c**	control
10^{-8}	0.072±0.004*	0.088±0.005	0.087±0.008*	0.036±0.009	3497±94*	4025±253
10^{-7}	0.070±0.004*	0.081±0.005	0.055±0.010*	0.026±0.006	3607±112*	3924±136
10^{-6}	0.074±0.004*	0.088±0.004	0.049±0.008	0.049±0.010	3576±68	3800±216
10^{-5}	0.070±0.002*	0.083±0.005	0.025±0.006*	0.039±0.006	3677±72	3844±112
10^{-4}	0.066±0.004	0.064±0.003	0.050±0.010*	0.097±0.010	3733±141	3992±178

* – significant difference from control, $p < 0.05$

Table S3. Effect of compounds **3a-c** on biomass (%), the level of metabolic activity, and the number of living cells in the bacterial biofilm of the *E. coli* CDC F-50

Concentration of **3a-c**	Intensity of biofilm formation, % of control	Metabolic activity of the biofilm, % of control	Number of viable cells, % of control
		**3a**	
10^{-8}	125	43	70*
10^{-7}	83	38*	63*
10^{-6}	108	96	66*
10^{-5}	115	24*	69*
10^{-4}	37*	34*	69*
		**3b**	
10^{-8}	67	222*	101
10^{-7}	70	224*	112*
10^{-6}	45*	148*	102
10^{-5}	82	130*	103
10^{-4}	28*	46*	101
		**3c**	
10^{-8}	48*	242*	87*

10^{-7}	54*	212*	92*
10^{-6}	55*	100	94
10^{-5}	50*	64*	96
10^{-4}	129	52*	94

* – significant difference from control, $p < 0.05$

Table S4. Effect of compounds **3a-c** on biomass, the level of metabolic activity, and the number of living cells in the bacterial biofilm of the *S. aureus* ATCC 6538-P FDA 209-P

Concentration of **3a-c**	Intensity of biofilm formation, OD 570 nm		Metabolic activity of the biofilm, OD 486 nm		Number of viable cells, Fluorescence intensity λ_{ex} = 485 ± 12 nm, λ_{em} = 520 ± 10 nm	
	**3a**	control	**3b**	control	**3c**	control
10^{-8}	0.216±0.014	0.236±0.018	0.024±0.005*	0.032±0.002	7694±140*	7289±134
10^{-7}	0.174±0.010*	0.230±0.030	0.025±0.001*	0.031±0.002	7384±104*	7754±148
10^{-6}	0.213±0.006	0.221±0.019	0.046±0.009*	0.090±0.005	7487±91	7529±185
10^{-5}	0.206±0.009*	0.233±0.012	0.050±0.008*	0.088±0.008	7511±104	7303±107
10^{-4}	0.217±0.010*	0.243±0.012	0.056±0.003*	0.068±0.006	7487±166	7482±109
	**3a**	control	**3b**	control	**3c**	control
10^{-8}	0.210±0.003*	0.229±0.004	0.020±0.002*	0.025±0.002	7371±121	7472±42
10^{-7}	0.210±0.009*	0.238±0.009	0.030±0.009	0.024±0.004	7591±167	7468±166
10^{-6}	0.190±0.007*	0.213±0.002	0.028±0.002*	0.036±0.005	7385±134	7290±102
10^{-5}	0.196±0.011	0.209±0.008	0.022±0.002*	0.033±0.005	7575±76	7493±113
10^{-4}	0.196±0.013*	0.167±0.005	0.047±0.002*	0.051±0.001	7507±169	7360±124
	**3a**	control	**3b**	control	**3c**	control
10^{-8}	0.095±0.006*	0.142±0.013	0.140±0.015*	0.177±0.017	5986±256	5724±323
10^{-7}	0.095±0.004*	0.111±0.005	0.147±0.008*	0.172±0.008	6351±109	6569±329
10^{-6}	0.089±0.004*	0.113±0.007	0.159±0.011	0.176±0.013	6092±381	6220±261
10^{-5}	0.090±0.004*	0.119±0.010	0.182±0.006	0.183±0.012	6229±486	6380±319
10^{-4}	0.085±0.004*	0.119±0.011	0.215±0.006*	0.274±0.011	6126±377	6566±501

* – significant difference from control, $p < 0.05$

Table S5. Effect of compounds **3a-c** on biomass (%), the level of metabolic activity, and the number of living cells in the bacterial biofilm of the *S. aureus* ATCC 6538-P FDA 209-P

Concentration of **3a-c**	Intensity of biofilm formation, % of control	Metabolic activity of the biofilm, % of control	Number of viable cells, % of control
	3a		
10^{-8}	89	75*	106*
10^{-7}	68*	81*	95*
10^{-6}	95	51*	99
10^{-5}	85*	57*	103
10^{-4}	86*	82*	100
	3b		
10^{-8}	89*	80*	99
10^{-7}	85*	125	102
10^{-6}	85*	78*	101
10^{-5}	91	67*	101
10^{-4}	126*	92*	102
	3c		
10^{-8}	45*	79*	105
10^{-7}	70*	85*	97
10^{-6}	57*	90	98
10^{-5}	53*	99	98
10^{-4}	45*	78*	93

* – significant difference from control, $p < 0.05$

Table S6 - Toxicity studies results of compounds **3a-c** using lux-biosensors

	Concentration of **3a-c**	*E. coli MG* 1655 (pRecA-lux)		*E. coli MG* 1655 (pAlkA-lux)		*E. coli MG* 1655 (pSoxS-lux)	*E. coli MG 1655* (pKatG-lux)	*E. coli MG 1655* (pIbpA-lux)	*V. aquamarinus* VKPM B-11245
		-S9	+S9	-S9	+S9				
3a	10^{-8}	1,06±0,09	1,06±0,04	1,18±0,22	1,28±0,13	0,93±0,02	0,96±0,06	1,02±0,07	0,00±0,00
	10^{-7}	1,02±0,08	1,03±0,01	1,14±0,01	1,27±0,01	1,01±0,06	0,91±0,01	0,96±0,07	0,00±0,00
	10^{-6}	1,14±0,09	0,91±0,02	1,49±0,11	1,29±0,15	1,16±0,08	1,02±0,10	1,15±0,03	0,00±0,00
	10^{-5}	1,06±0,06	1,06±0,01	1,53±0,20	1,19±0,02	1,37±0,06	1,14±0,02	1,03±0,05	43,38±0,00*
	10^{-4}	1,99±0,17	1,09±0,03	2,14±0,26*	1,63±0,14	1,63±0,11	2,06±0,10*	1,90±0,11	12,23±0,00
3b	10^{-8}	1,98±0,16	1,07±0,01	2,41±0,22*	1,61±0,04	1,52±0,15	1,81±0,14	2,10±0,20*	0,00±0,00
	10^{-7}	1,13±0,07	1,07±0,03	2,45±0,28*	2,90±0,20*	0,99±0,09	1,07±0,09	1,14±0,08	0,00±0,00
	10^{-6}	0,97±0,04	1,06±0,02	1,75±0,08	1,88±0,04	1,21±0,05	1,07±0,04	1,09±0,07	30,01±0,01*
	10^{-5}	1,33±0,09	1,10±0,02	1,92±0,07	1,74±0,08	1,55±0,18	1,48±0,13	1,25±0,11	44,18±0,01*
	10^{-4}	2,57±0,10*	1,13±0,07	2,70±0,11*	1,91±0,09	1,84±0,08	2,50±0,07*	2,52±0,08*	0,00±0,00
3c	10^{-8}	1,63±0,11	1,06±0,01	1,95±0,18	1,68±0,06	1,12±0,08	1,59±0,13	1,73±0,09	0,00±0,00
	10^{-7}	1,95±0,03	1,09±0,02	1,68±0,23	2,19±0,62*	1,16±0,06	1,00±0,03	0,98±0,04	11,90±0,02
	10^{-6}	1,29±0,19	1,01±0,02	1,82±0,30	1,58±0,15	1,40±0,24	1,39±0,20	1,10±0,18	5,34±0,05
	10^{-5}	1,33±0,04	1,06±0,03	1,77±0,08	1,62±0,10	1,38±0,04	1,38±0,05	1,33±0,04	42,80±0,01*
	10^{-4}	1,99±0,03	1,02±0,03	2,34±0,11*	2,35±0,15*	1,47±0,08	2,11±0,04*	1,86±0,05	5,76±0,03

* – significant difference from control, $p < 0.05$

5. Molecular docking

Table S7. Optimized geometry of the compound **3a** in water (CPCM model).

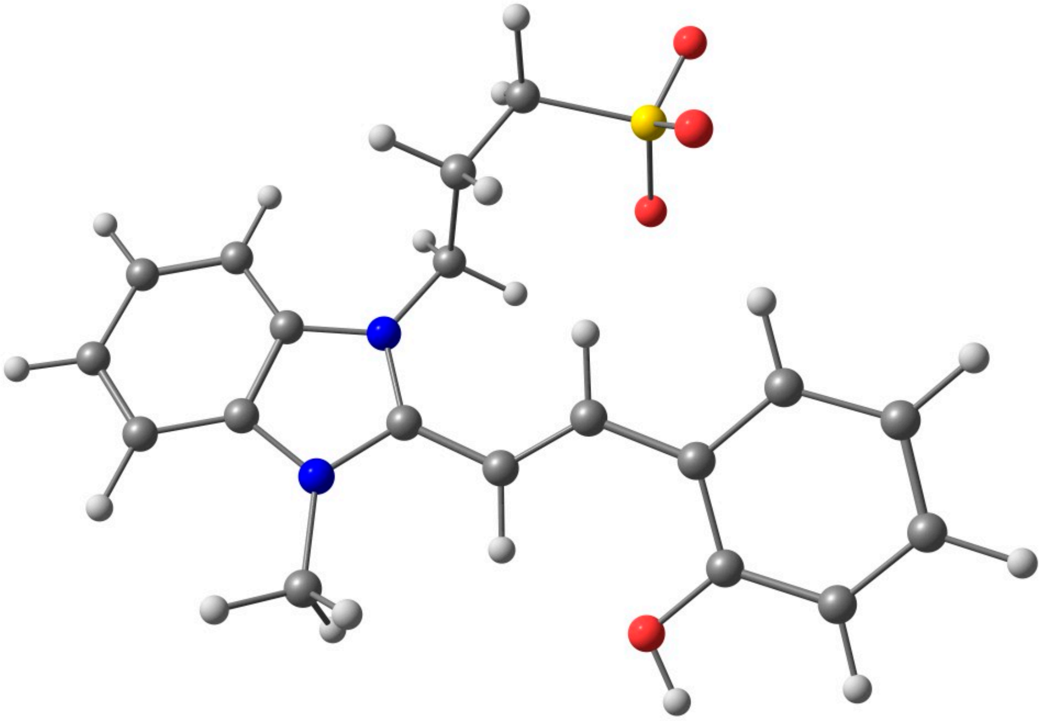

Atom	X	Y	Z
**S**	3.52621275995527	14.44430346511404	4.24004833165186
**O**	8.73434814788154	10.20085487136460	5.66289756432509
**N**	5.99915064789088	8.70842898018807	2.07226187536478
**O**	4.94751546947176	14.53386325418170	3.90547145412511
**O**	2.86770275502217	15.74674441408378	4.27718224195650
**O**	3.27827873323134	13.64434880695243	5.44000353244480
**C**	7.92612130610578	12.47036998759839	8.39597616482778
**H**	8.36476360259313	12.81210910636073	9.32660155621320
**C**	5.23881762771376	8.65559148414057	0.92381733025617
**C**	6.82000148219711	11.56741510211471	5.98345043061142
**C**	8.61137542506760	11.55060871948234	7.62173531584182
**N**	5.00008604164040	10.64643890140307	1.90285484421856
**C**	8.07366056148010	11.09508262703878	6.42320391228997
**C**	6.15337146833572	12.50119615756531	6.79377052716708
**H**	5.19267815139086	12.87355819711733	6.45097576876109
**C**	6.68845847353255	12.95203706481544	7.98301901790251
**C**	6.15499071566364	11.17246067590779	4.76274491915416
**H**	5.20130931126496	11.67067496540924	4.62535345475456
**C**	4.59623809087622	9.89023683977129	0.81909888557204
**C**	6.55849142832553	10.27007635909134	3.84512785288618
**H**	7.47214371336102	9.71513609177083	3.99849634536087
**C**	5.84535606878816	9.91406024143155	2.65466925060817
**C**	5.05681082497399	7.65696546593108	-0.02249459103829
**H**	5.55633697357721	6.69967256395818	0.05291104322243
**C**	3.56603766297101	9.18787278987674	-1.18273258184273
**H**	2.91044159032034	9.37253487234777	-2.02503444634840
**C**	4.20750672421242	7.94740098731487	-1.07535106549394
**H**	4.03633102963742	7.19669578797739	-1.83733221719941
**C**	3.74752590521120	10.18327272586563	-0.23888616120096
**H**	3.24780924364097	11.13958382101187	-0.32430890879107
**C**	6.81892092082804	7.62970020211077	2.58539759582416

**H**	6.51928326827168	6.70829999569658	2.09293770330976
**H**	6.66258870708845	7.52517613798585	3.65739948767744
**H**	7.87292418439579	7.82228911490193	2.38141581412221
**C**	2.74237428699189	13.55872129728600	2.90233980162782
**H**	1.67508052071176	13.57680793305798	3.12955684103037
**H**	2.90648160243112	14.14719888140675	1.99613422563486
**C**	4.59785780262107	12.02782510133918	2.10367245319446
**H**	4.59715046555716	12.50491763484636	1.12138073669917
**H**	5.35521650196923	12.52585895798993	2.70424163396683
**C**	3.21857671148259	12.12683689711317	2.73733331938637
**H**	3.21204561891656	11.61984130298662	3.70596592388485
**H**	2.50270425787823	11.59720844255102	2.10326875135927
**H**	9.57778913726386	11.17242166477591	7.93983639685522
**H**	9.57228714205913	9.96930019204942	6.08227401536851
**H**	6.15065513119914	13.67399397371525	8.58529084345726

Table S8. Optimized geometry of the compound **3b** in water (CPCM model).

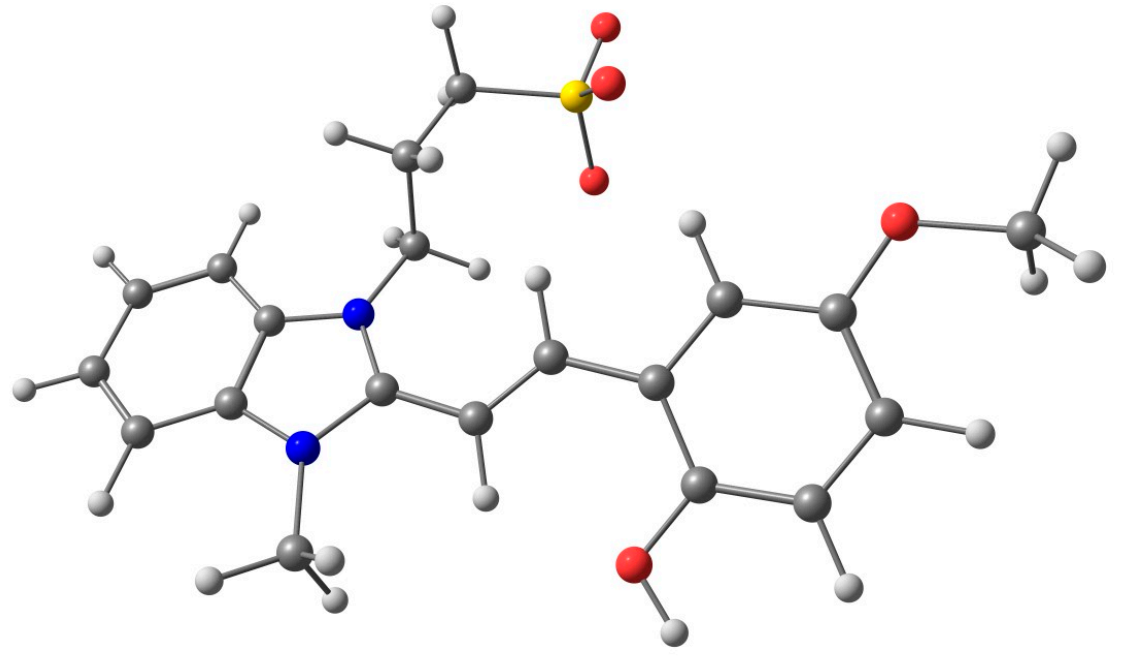

Atom	X	Y	Z
**S**	3.42463172474323	4.18986272884864	4.18986272884864
**O**	8.56116753299085	5.77384785354637	5.77384785354637
**N**	5.99010370356532	2.04615021426931	2.04615021426931
**O**	4.85328149380051	3.89561064839118	3.89561064839118
**O**	2.75054330466793	4.22635055351917	4.22635055351917
**O**	3.15325877605525	5.37207797060181	5.37207797060181
**C**	7.69251163914607	8.46466482129890	8.46466482129890
**H**	8.11144974606638	9.40706929360752	9.40706929360752
**C**	5.27642425396268	0.86770420926971	0.86770420926971
**C**	6.67028755890198	6.01051031680671	6.01051031680671
**C**	8.37900206890171	7.71574725368646	7.71574725368646
**N**	4.99867419483790	1.85640270683896	1.85640270683896
**C**	7.89118294227879	6.50177925376940	6.50177925376940
**C**	5.99195894680311	6.78080777574101	6.78080777574101
**H**	5.05479048634973	6.40848429670628	6.40848429670628
**C**	6.48533346002163	7.99427216222391	7.99427216222391
**C**	6.04519847400997	4.76205909433509	4.76205909433509
**H**	5.10571878439736	4.58934940106768	4.58934940106768

**C**	4.63927627623116	0.74958256560591	0.74958256560591
**C**	6.47540350809795	3.85761806650510	3.85761806650510
**H**	7.37538325261046	4.04859888522757	4.04859888522757
**C**	5.81239822646195	2.63440433935043	2.63440433935043
**C**	5.13184004565786	-0.09577393319208	-0.09577393319208
**H**	5.62645645963941	-0.01020987774093	-0.01020987774093
**C**	3.69020167902799	-1.29932847756657	-1.29932847756657
**H**	3.06931409948152	-2.16549433975784	-2.16549433975784
**C**	4.32607596552969	-1.17891327721036	-1.17891327721036
**H**	4.18524908220501	-1.95489702870395	-1.95489702870395
**C**	3.83425535753217	-0.33865408141330	-0.33865408141330
**H**	3.33924742535501	-0.43358610851740	-0.43358610851740
**C**	6.78732514021330	2.58333825827608	2.58333825827608
**H**	6.53634866726222	2.04350286202491	2.04350286202491
**H**	6.55875240009041	3.63847398218123	3.63847398218123
**H**	7.84995734127755	2.45891161091663	2.45891161091663
**C**	2.68730614012975	2.81991903741532	2.81991903741532
**H**	1.61395024608382	3.01724086890403	3.01724086890403
**H**	2.87118155892899	1.92578444175327	1.92578444175327
**C**	4.58028061396527	2.05917730548119	2.05917730548119
**H**	4.60450475472539	1.08215036503804	1.08215036503804
**H**	5.31574075081588	2.68658711115947	2.68658711115947
**C**	3.18142015505485	2.65159504457646	2.65159504457646
**H**	3.14978471691287	3.61329284666119	3.61329284666119
**H**	2.49039894521818	1.98894254021772	1.98894254021772
**H**	9.31968080771416	8.08607329494656	8.08607329494656
**H**	9.36856355640709	6.23610569351994	6.23610569351994
**O**	5.73760718152811	8.64598260785388	8.64598260785388
**C**	6.22210249304642	9.88693699875834	9.88693699875834
**H**	6.31152276029425	10.61465153065273	10.61465153065273
**H**	7.19343496991700	9.77345423769576	9.77345423769576
**H**	5.49109282008361	10.24190053885231	10.24190053885231

Table S9. Optimized geometry of the compound **3c** in water (CPCM model).

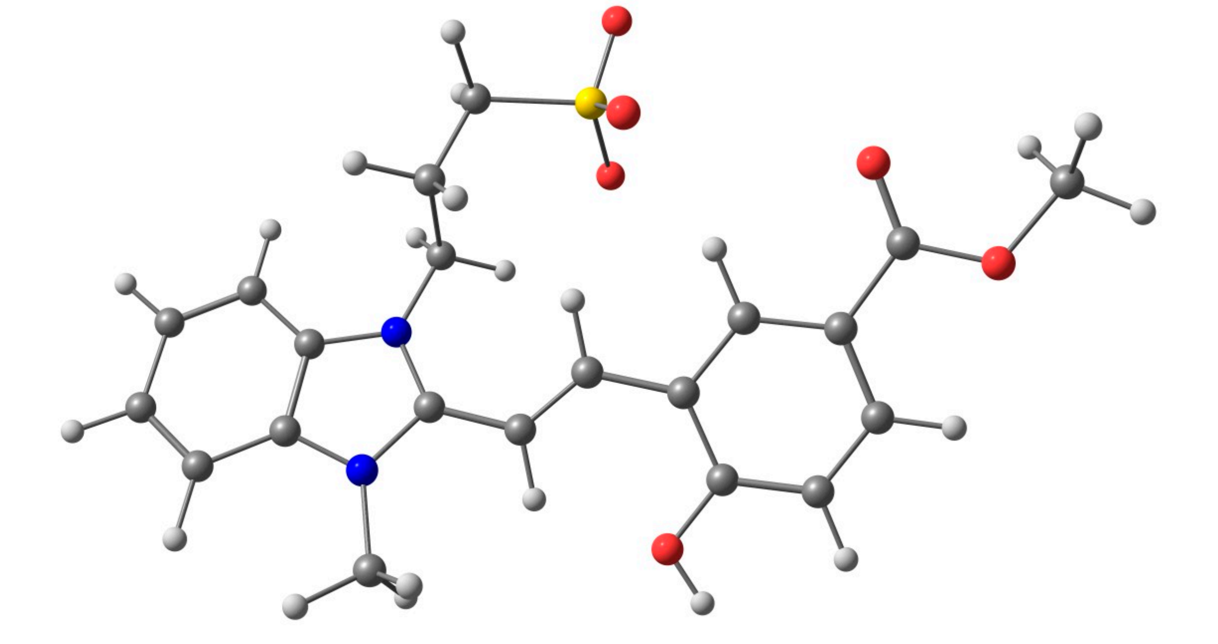

Atom	X	Y	Z
**S**	3.39328216112507	14.33889771807633	4.23714862959112
**O**	8.48336332233326	10.01033422489584	5.84558006006646

**N**	5.98557677735860	8.71565355554632	2.06622539326862
**O**	4.82463060370363	14.47646033142018	3.96698614000061
**O**	2.70524128089585	15.62293422456825	4.32745794940925
**O**	3.11834863732414	13.45963292012482	5.37406998671366
**C**	7.80519416524886	12.47892032342169	8.42219077067840
**H**	8.23375988502467	12.82325625877138	9.35420212615226
**C**	5.29688084506607	8.67483812224689	0.87334157092343
**C**	6.71058939247433	11.57650831137619	5.99792275771236
**C**	8.41630540351315	11.45749058919163	7.72820030878029
**N**	5.02110050912858	10.66527433888199	1.84259781728399
**C**	7.88822150751950	11.00046029587696	6.52347073803341
**C**	6.11249918800606	12.60675233872656	6.72935875371215
**H**	5.20704034750165	13.05802553396120	6.33703707730684
**C**	6.64123112003991	13.06775254554920	7.92434872457426
**C**	6.06746942832701	11.19925662851213	4.75638510123867
**H**	5.15623530588584	11.75790320578614	4.57329836019502
**C**	4.67641926878109	9.91717596147107	0.73386087013762
**C**	6.46318921945530	10.26243361858759	3.87231447863714
**H**	7.33910224645652	9.66662737847958	4.08094706728787
**C**	5.81055036739217	9.92196956476754	2.64207209877749
**C**	5.16089619408288	7.68149173372360	-0.08673541258089
**H**	5.64306066458670	6.71780169525956	0.01423669037620
**C**	3.76019777584710	9.23418975182767	-1.32844517417812
**H**	3.15868108244145	9.42890434935703	-2.20798054530343
**C**	4.38053332235317	7.98584652366403	-1.18749114762633
**H**	4.24730209786344	7.23992196442757	-1.96160530619822
**C**	3.89571506212110	10.22394053453238	-0.37170004364647
**H**	3.41311471248637	11.18647248503652	-0.48180839764434
**C**	6.76256700339146	7.62586092579361	2.62196489728315
**H**	6.49412786300476	6.71262942107520	2.09770775240787
**H**	6.53066308028888	7.50762011594483	3.67874447671011
**H**	7.82902962918527	7.81541601618341	2.49451637030979
**C**	2.68377514683973	13.51873678314420	2.81876394297113
**H**	1.60663377263943	13.51673388313505	2.99407011077590
**H**	2.88607807246497	14.15226715039135	1.95157994266496
**C**	4.59045350486226	12.03853068102298	2.03838743799579
**H**	4.62240298226440	12.52516517219476	1.06169234728480
**H**	5.31341926194605	12.54371986893494	2.67429883947598
**C**	3.18335503247240	12.10029978231727	2.61360566317311
**H**	3.14226945196698	11.55471970557333	3.56005984583823
**H**	2.50293980468401	11.58943577360457	1.92728619856634
**H**	9.32051078469707	10.99699705584256	8.11225879156138
**H**	9.27151479176083	9.71059859012375	6.31688750046921
**C**	5.95916933299877	14.17562213860668	8.62486464865533
**O**	4.96974137423805	14.74085437933743	8.21489974147434
**O**	6.55619118968082	14.49714930849483	9.77195163773774
**C**	5.96491248996335	15.56709777315653	10.50930376980376
**H**	5.96842857980575	16.48361617065205	9.91857395528057
**H**	4.94053204227536	15.31754156397189	10.78739083775683
**H**	6.57961685422568	15.68704750043023	11.39780779212409

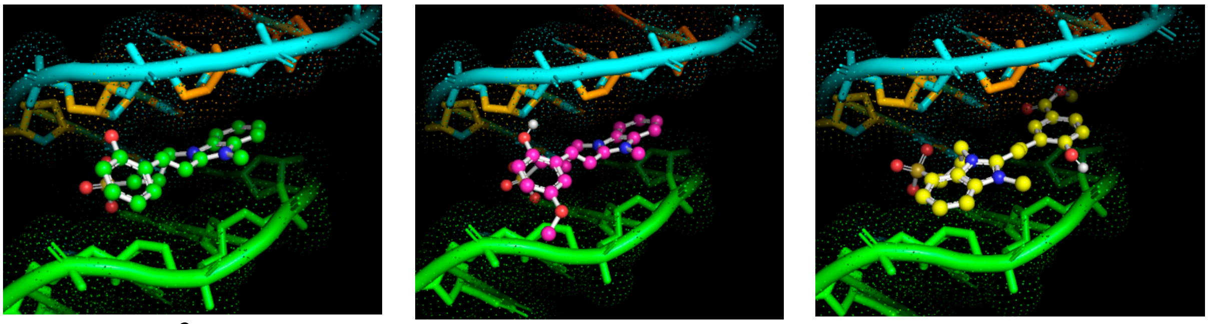

3a **3b** **3c**

Figure S30. Results of molecular docking for **3a-c** with DNA in minor groove modes

6. Fluorescent microscopy

Biofilm formation for bioimaging experiment using fluorescence microscopy. *S. aureus* 6538-P FDA 209-P and *E. coli* CDC F-50 was cultivated in Erlenmeyer flasks with LB medium in a gyratory shaker incubator, Innova 40R (New Brunswick, Canada) for 24 h at 37 °C. The daily culture suspension was diluted with a nutrient medium to a turbidity of 1 McFarland unit (3×10^8 cells/mL) measured on a DEN-1 densitometer (BioSan, Latvia). The resulting suspension was incubated for 3/24 h at 37 °C in LB medium with the addition of the test substance, the final concentration of which was 10^{-4} M. Cultivation was carried out in 100 ml glass laboratory beakers in which slides were placed at an angle. The biofilm formed on the glasses was used for further studies.

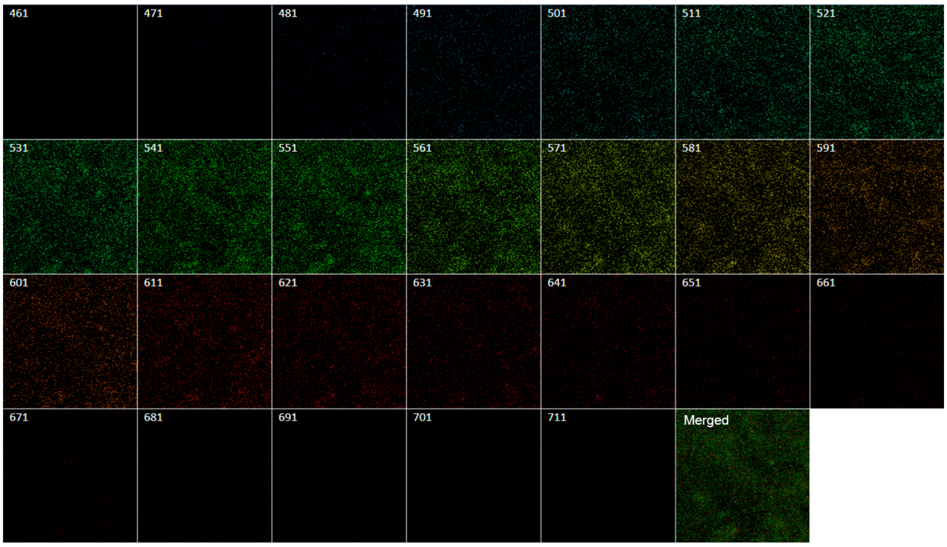

Figure S31. Split image of lambda-stack obtained as a result of bioimaging experiment using biofilms of *E. coli* CDC-F50 treated by solution of dye **3c** during 3 hours (10^{-4} M, excitation by laser with $\lambda = 458$ nm)

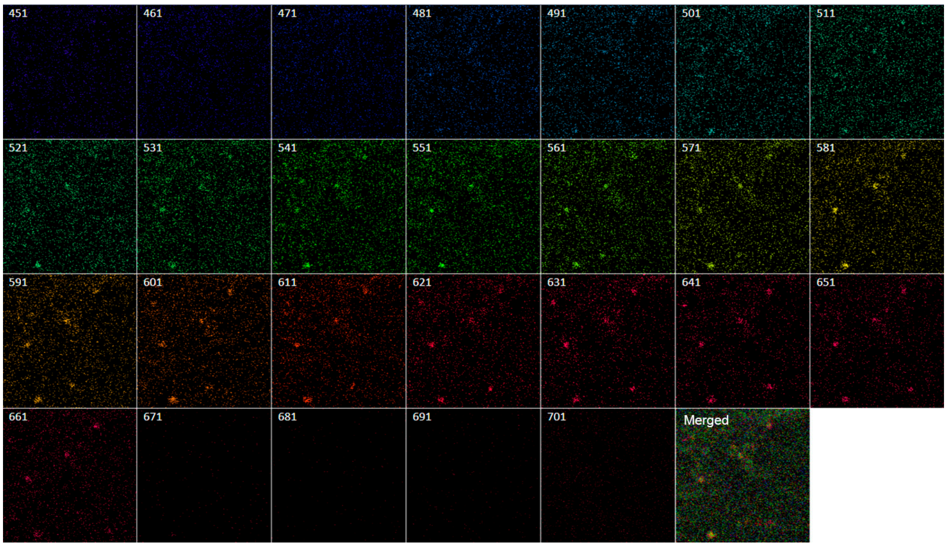

Figure S32. Split image of lambda-stack obtained as a result of bioimaging experiment using biofilms of *E. coli* CDC-F50 treated by solution of dye **3c** during 3 hours(10^{-4} M, excitation in multiphoton mode with $\lambda = 725$ nm)

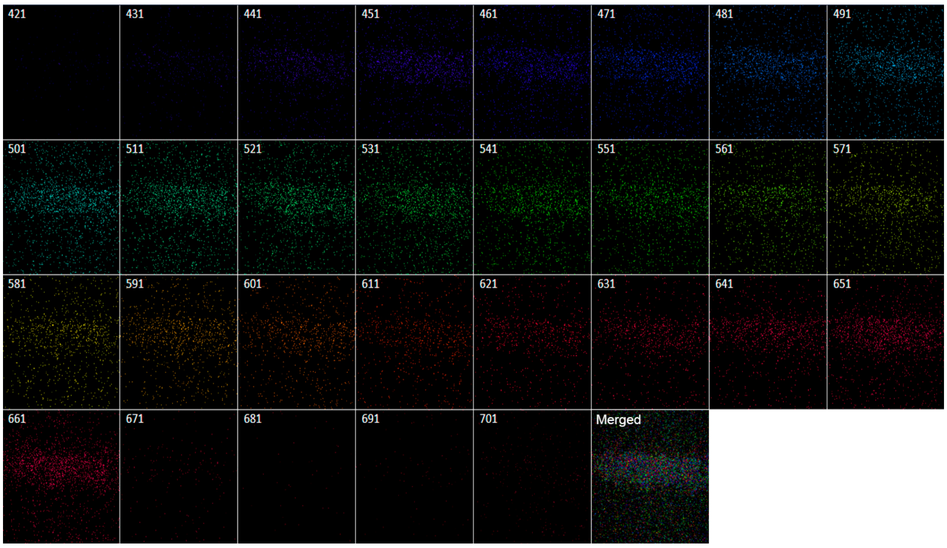

Figure S33. Split image of lambda-stack obtained as a result of bioimaging experiment using biofilms of *E. coli* CDC-F50 treated by solution of dye **3c** during 24 hours (10^{-4} M, excitation in multiphoton mode with $\lambda = 725$ nm)

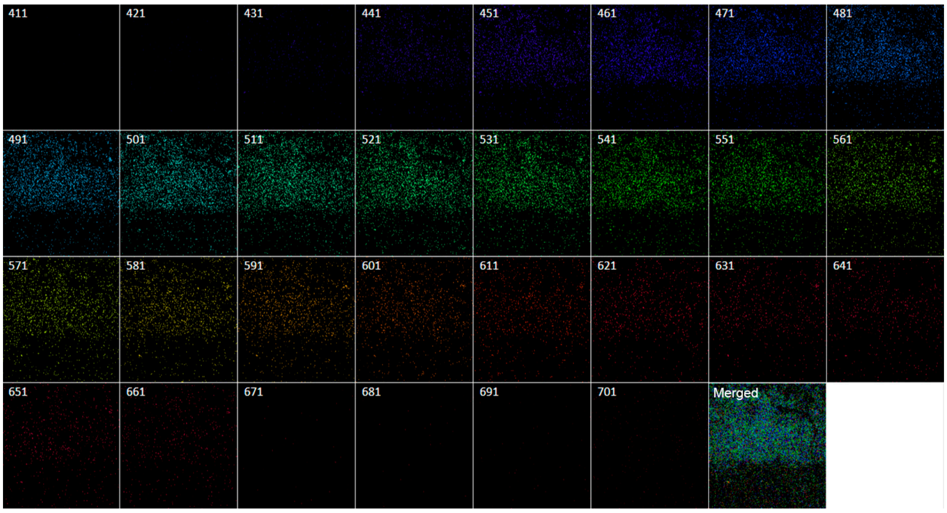

Figure S34. Split image of lambda-stack obtained as a result of bioimaging experiment using biofilms of *S. aureus* ATCC 6538-P FDA 209-P treated by solution of dye **3c** during 24 hours (10^{-4} M, excitation in multiphoton mode with $\lambda = 725$ nm)

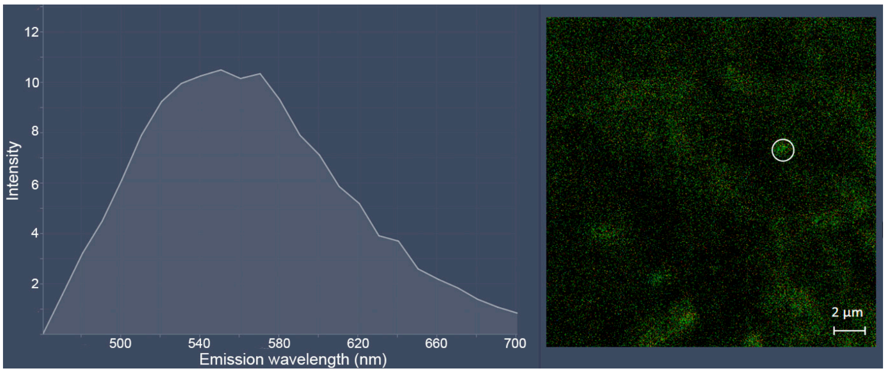

Figure S35. Image of lambda-stack obtained as a result of bioimaging experiment using biofilms of *E. coli* CDC-F50 treated by solution of dye **3c** during 3 hours (10^{-4} M, excitation by laser with $\lambda = 458$ nm).

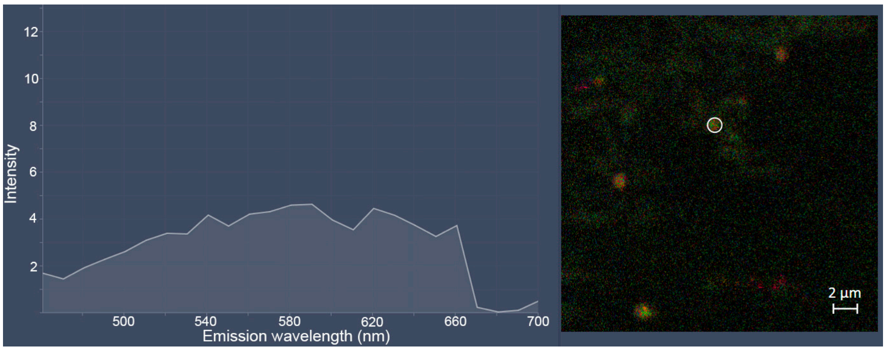

Figure S36. Image of lambda-stack obtained as a result of bioimaging experiment using biofilms of *E. coli* CDC-F50 treated by solution of dye **3c** during 3 hours (10^{-4} M, excitation in multiphoton mode with $\lambda = 725$ nm).

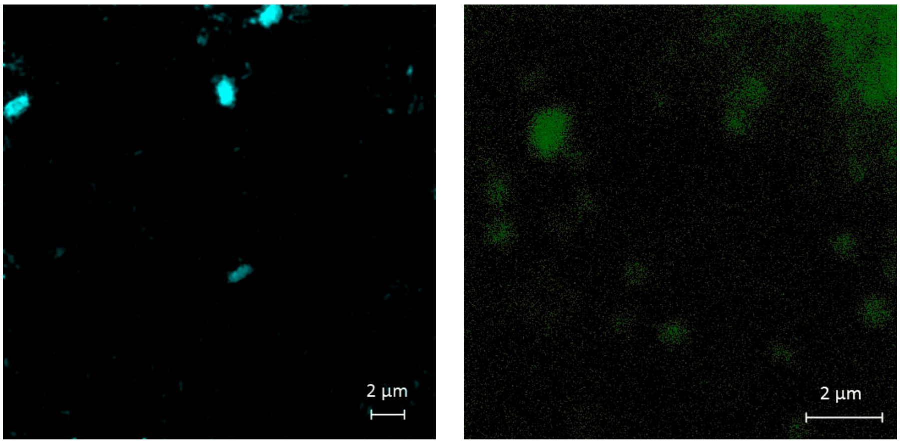

(a) **(b)**

Figure S37. Crop images of bacterial cells in biofilms of *E. coli* CDC-F50 **(a)** and *S. aureus* ATCC 6538-P FDA 209-P **(b)** treated by solution of dye **3c** during 24 hours.

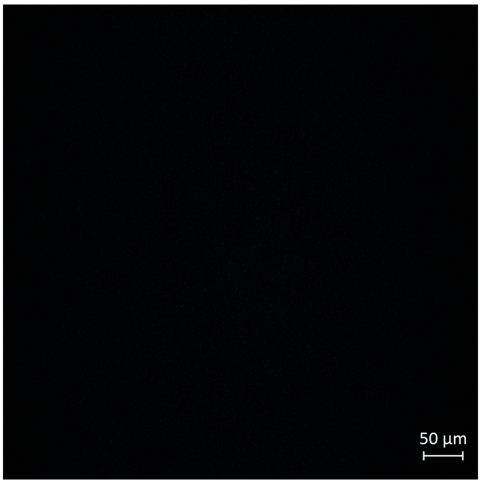

Figure S38. Image of control bioimaging experiment using biofilms of *E. coli* CDC-F50 without dye **3c** (excitation by laser with $\lambda = 458$ nm)

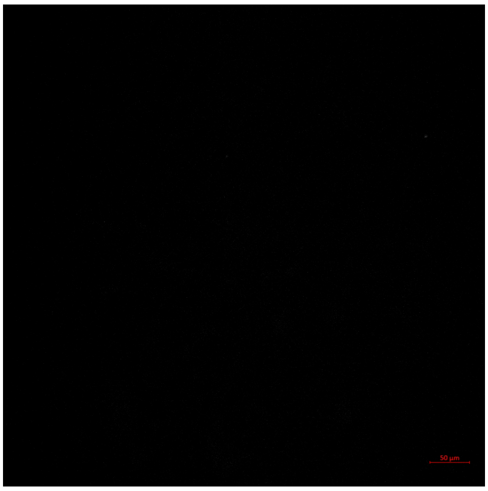

Figure S39. Image of control bioimaging experiment using biofilms of *E. coli* CDC-F50 without dye **3c** (excitation in multiphoton mode with $\lambda = 725$ nm)
